# Supplementary material for: Bayesian uncertainty analysis for complex systems biology models: emulation, global parameter searches and evaluation of gene functions
Source: BMC Syst Biol. 2018 Jan 2;12:1. doi: 10.1186/s12918-017-0484-3 (PMC5748965; doi:10.1186/s12918-017-0484-3)
Supplement: Supplementary file 2 — This file gives the details of the full emulator structure used in the wave 1 emulators, described in the subsection entitled “Bayesian emulation of the Arabidopsis model”, and gives the dimensions or units of all the rate constants. (PDF 1444 kb) [file 12918_2017_484_MOESM2_ESM.pdf]

METHODOLOGY ARTICLE

Supplementary Material for the article Bayesian uncertainty analysis for complex systems biology models: emulation, global parameter searches and evaluation of gene functions.

Ian Vernon[1*], Junli Liu[2†], Michael Goldstein[1], James Rowe[3], Jen Topping[2] and Keith Lindsey[2^]

*Correspondence:
i.r.vernon@durham.co.uk
[1]Department of Mathematical Sciences, Durham University, South Road, DH1 3LE, Durham, UK
Full list of author information is available at the end of the article
†Joint corresponding author:
junli.liu@durham.ac.uk,
^michael.goldstein@durham.ac.uk,
J.H.Rowe@sheffield.ac.uk,
j.f.topping@durham.ac.uk,
keith.lindsey@durham.ac.uk.

Summary

Here we provide more details as to the structure of the wave 1 emulators, showing their full regression structure and summarising the active inputs for each of the 13 outputs that were emulated in the first wave.

Emulator Structure for the First Wave.

Table 1 gives the active inputs x_{A_i} for each of the 13 outputs emulated at wave 1. We can see for example that outputs that involve feeding Auxin (denoted by an "fa" suffix), feeding ethylene ("fe") or feeding cytokinin ("fc") possess the correct active input related to that kind of feeding: one of $k_{1vauxin}$, k_{1vCK} or k_{1veth} respectively.

Tables 2-14 give the chosen form for the regression part of the emulator in terms of the deterministic functions $g_{ij}(x_{A_i})$, for each of the 13 outputs that were emulated in wave 1. The tables show the linear model output from the stepwise selection function step(). For brevity, the specific inputs shown in these tables are the numerators from the ratio of inputs given in Table 2 of the main paper. Note that the colon ":" used in the left column of these tables represents the interaction term between the two stated inputs. So for example, $k2 : k1$ represents the term in the regression of the form $\beta_j k_2 k_1$, and the estimated value and standard error of the fitted constant β_j is given in the second and third column respectively.

Table 15 gives the dimensions or units of the original rate parameters that feature in Table 1 of the main article.

Emulator Diagnostics

Tables 16 and 17 give 200 run emulator prediction diagnostics for the 13 outputs used in wave 1. The 200 emulator prediction intervals $\mathrm{E}_{D_i}(f_i(x)) \pm 2\sqrt{\mathrm{Var}_{D_i}(f_i(x))}$ are plotted (y-axis) against the true values of the 200 runs (x-axis). The diagonal dashed line gives the line $y = x$ and represents a perfect prediction: if we assume normality, for example, we would expect approximately 95% of the intervals to lie on

this line. If we do not want to assume normality, which we do not have to in a Bayes linear context, we would expect fewer than this: just assuming unimodality would suggest 95% within ±3 sigma for example. Most importantly, the target interval that we are trying to match the model output to (shown as the error bars in figures 7 and 10 in the main article) is given here by the three horizontal lines. We see for several outputs that the emulators are reasonably accurate and behaving well, for example, outputs CK_{wt}, $Auxin_{fa}$, EF_{fe}, CK_{fa}, CK_{fc}, $PLSp_{fa}$, $PLSp_{fe}$. For the other outputs, the emulators are less accurate but the diagnostics are still safe (see for example $Auxin_{fe}$), in that although there are a small number of outliers where the prediction interval is far from the true value, for each of these prediction intervals the emulator's estimate is actually closer to the observed target interval than the actual true value is. Hence emulators of this form are cautious: they should not incorrectly rule out acceptable runs, instead they will sometimes fail to rule out unacceptable runs. This is all we need to be able to safely rule out parts of the input space, and progress to the next wave. This is in fact an important feature of emulator diagnostics in a history matching context, as we can place less stringent demands on the emulators, and is seen throughout the remaining diagnostic plots.

Table 18 shows the progression of the emulators for output ET_{fe} (left column) and output $PLSp_{fa}$ (right column) through waves 1 to 4 (top to bottom row). The horizontal lines again give the target interval. For ET_{fe} (left column), we see a notable increase in emulator accuracy judging by the size of the prediction intervals, going from wave 1 to 2, and by wave 4 (noting the zoomed in scale on both axes), the vast majority of the points are now within the target range. The emulators for $PLSp_{fa}$ (right column) are less accurate but still show a similar progression: there is increasing emulator accuracy across the waves, noting again the zoomed in y-axis, leading to the majority of wave 4 points being in the target range.

For completeness we give also give the full diagnostics for waves 2-4 in tables 19 to 27, showing again cautious emulators that allow the identification of runs lying in the target range. A notable output which was difficult to emulate accurately was $Auxin_{fc}$, but the wave 4 emulators were at this stage informative enough to generate large numbers of points that were acceptable matches to all other outputs, and a substantial proportion of these also matched $Auxin_{fc}$ (this corresponds to step 7 of the history matching algorithm, on page 9 of the main article, see also [1]).

Author details

[1]Department of Mathematical Sciences, Durham University, South Road, DH1 3LE, Durham, UK. [2]Department of Biosciences, Durham University, South Road, DH1 3LE, Durham, UK. [3]Department of Biosciences, Durham University, South Road, Durham, DH1 3LE, UK, current address: Department of Molecular Biology and Biotechnology, University of Sheffield, Firth Court, Western Bank, S10 2TN, Sheffield, UK.

References

1. Andrianakis, I., McCreesh, N., Vernon, I., McKinley, T.J., Oakley, J.E., Nsubuga, R., Goldstein, M., White, R.G.: History matching of a high dimensional individual based hiv transmission model. Journal of Uncertainty Quantification **to appear** (2016)

	Auxin_mu	Auxin_fa	Auxin_fe	Auxin_mufe	ET_fa	ET_fe	CK_fa	CK_fe	CK_fc	PLSp_fa	PLSp_fe	Auxin_wt	CK_wt
k1	1	1	1		1	1	1		1	1		1	1
k2	1	1	1	1	1	1	1		1	1		1	1
k2a	1	1	1				1	1	1	1	1	1	1
k2b	1	1	1				1	1			1	1	
k2c	1	1	1					1		1	1	1	1
k3	1			1	1	1	1	1	1	1		1	1
k3a				1		1	1	1	1	1		1	1
k5	1		1							1	1	1	1
k6a	1		1					1		1	1	1	
k7	1		1								1		
k9	1		1		1			1					
k10a					1								
k11				1									
k12a	1	1		1	1	1				1	1		
k13	1	1	1	1			1	1	1	1	1	1	1
k15				1								1	
k16a	1	1		1					1				
k17		1		1	1	1	1		1	1		1	1
k18			1		1	1	1	1	1	1			1
k19		1	1	1	1	1		1				1	1
k1vauxin		1			1		1			1			
k1vCK									1				
k1veth			1	1		1		1			1		

Table 1 The active inputs, denoted as a 1, chosen for each of the 13 outputs emulated in wave 1.

	Estimate	Std. Error	t value	Pr(>|t|)
(Intercept)	-1.73	0.05	-35.27	0.00
k17	1.59	0.03	48.63	0.00
k3	-1.49	0.03	-59.57	0.00
k3a	-1.13	0.03	-39.36	0.00
k2	0.95	0.04	26.91	0.00
k13	-0.24	0.04	-5.65	0.00
k2c	-0.09	0.01	-8.04	0.00
k1	0.57	0.04	14.23	0.00
k2a	0.53	0.04	13.90	0.00
k6a	0.06	0.01	6.20	0.00
k15	-0.18	0.03	-5.66	0.00
k2b	0.21	0.03	6.57	0.00
k19	0.04	0.02	2.39	0.02
k5	-0.07	0.03	-2.63	0.01
I(k3a^2)	-0.48	0.05	-10.00	0.00
I(k13^2)	0.29	0.05	5.84	0.00
I(k2^2)	0.41	0.05	8.57	0.00
I(k3^2)	-0.40	0.05	-8.30	0.00
I(k17^2)	-0.37	0.05	-7.55	0.00
I(k2a^2)	0.38	0.06	6.71	0.00
I(k15^3)	0.04	0.01	3.00	0.00
k3:k3a	0.87	0.04	20.17	0.00
k17:k3	-0.81	0.04	-18.41	0.00
k17:k3a	0.83	0.04	18.89	0.00
k13:k2a	-0.64	0.05	-12.80	0.00
k17:k2	-0.53	0.04	-12.24	0.00
k13:k2c	0.14	0.02	9.40	0.00
k2:k1	-0.28	0.05	-5.65	0.00
k13:k2b	-0.18	0.03	-6.07	0.00
k3a:k13	0.19	0.03	6.46	0.00
k2a:I(k13^2)	0.19	0.04	5.14	0.00
k2c:k2a	-0.12	0.02	-7.30	0.00
k13:k6a	-0.05	0.01	-5.35	0.00
k2:k13	0.12	0.03	4.40	0.00
k2c:I(k13^2)	-0.06	0.01	-4.61	0.00
k13:k1	0.12	0.03	4.21	0.00
k1:k2b	-0.13	0.04	-3.17	0.00
k2:k2a	-0.27	0.05	-5.56	0.00
k17:k1	-0.15	0.04	-3.48	0.00
k2:k15	0.09	0.03	3.20	0.00
k13:I(k2a^2)	-0.20	0.06	-3.58	0.00
k17:k19	-0.11	0.03	-3.87	0.00
k1:I(k17^2)	-0.31	0.08	-3.64	0.00
k1:k2a	-0.25	0.05	-4.82	0.00
k13:I(k13^2)	-0.09	0.03	-3.18	0.00
k2:k2c	0.04	0.01	3.08	0.00
k3a:k2a	-0.12	0.04	-2.69	0.01
k17:k13	-0.09	0.03	-2.98	0.00
k2a:k2b	0.13	0.04	2.90	0.00
k2b:k19	-0.08	0.03	-2.82	0.00
k17:k3:k3a	-0.35	0.08	-4.60	0.00
k13:k2c:k2a	0.09	0.02	5.42	0.00
k2:k13:k1	-0.19	0.05	-3.84	0.00
k17:k2:k1	0.26	0.08	3.51	0.00
k13:k1:k2a	0.15	0.05	3.08	0.00
k2:k1:k2a	0.23	0.07	3.17	0.00
k2:k13:k2a	0.18	0.05	3.56	0.00

Table 2 The structure of the wave 1 emulator regression terms, for output Auxin-wt

	Estimate	Std. Error	t value	Pr(>|t|)
(Intercept)	-1.25	0.03	-43.02	0.00
k19	-2.32	0.01	-184.53	0.00
k18	1.36	0.03	51.02	0.00
k3	1.02	0.02	46.49	0.00
k17	-0.86	0.02	-39.21	0.00
k13	0.25	0.02	10.04	0.00
k3a	0.62	0.02	29.33	0.00
k2	-0.39	0.03	-14.61	0.00
k2c	0.04	0.01	4.87	0.00
k2a	-0.36	0.03	-13.02	0.00
k1	-0.26	0.02	-14.14	0.00
k5	0.03	0.02	1.99	0.05
I(k18^2)	-0.38	0.01	-27.12	0.00
I(k13^2)	-0.10	0.02	-5.96	0.00
I(k2^2)	-0.31	0.04	-8.55	0.00
I(k3a^2)	0.20	0.04	5.55	0.00
I(k2a^2)	-0.20	0.04	-5.42	0.00
k18:k3	-0.48	0.02	-24.96	0.00
k18:k17	0.39	0.02	17.73	0.00
k3:k17	0.73	0.03	21.44	0.00
k3:k3a	-0.62	0.03	-18.29	0.00
k13:k2a	0.43	0.03	12.76	0.00
k18:k3a	-0.26	0.02	-13.46	0.00
k18:k2	0.36	0.03	11.65	0.00
k18:k1	0.16	0.02	8.52	0.00
k13:k2c	-0.08	0.01	-8.11	0.00
k13:k3a	-0.13	0.02	-6.69	0.00
k18:k2a	0.15	0.02	6.66	0.00
k3:k13	-0.11	0.02	-5.94	0.00
k2a:I(k13^2)	-0.14	0.02	-5.45	0.00
k18:k13	-0.07	0.01	-5.53	0.00
k3:k2	0.16	0.03	5.67	0.00
k3a:k2a	0.21	0.03	6.25	0.00
k18:I(k2^2)	0.17	0.04	4.71	0.00
k2c:I(k13^2)	0.03	0.01	4.37	0.00
k2c:k2a	0.06	0.01	5.59	0.00
k13:I(k2a^2)	0.15	0.04	4.17	0.00
k3:k2a	0.18	0.03	5.22	0.00
k17:k3a	-0.24	0.03	-7.27	0.00
k3:k1	0.11	0.03	3.78	0.00
k3a:k2	0.10	0.03	3.45	0.00
k19:k18	0.04	0.01	3.48	0.00
k2:I(k18^2)	-0.08	0.02	-3.27	0.00
k2:k2a	0.09	0.03	3.14	0.00
k2:I(k13^2)	-0.04	0.01	-2.95	0.00
k17:k13	0.03	0.02	1.22	0.22
k18:I(k3a^2)	-0.11	0.04	-2.95	0.00
k18:k3:k17	-0.28	0.03	-8.32	0.00
k18:k3:k3a	0.29	0.03	8.74	0.00
k18:k13:k2a	-0.11	0.02	-4.70	0.00
k13:k3a:k2a	-0.13	0.03	-3.91	0.00
k13:k2c:k2a	-0.05	0.01	-4.91	0.00
k18:k17:k3a	0.26	0.03	7.93	0.00
k18:k17:k13	0.07	0.02	3.23	0.00
k3:k13:k2a	-0.10	0.03	-2.97	0.00

Table 3 The structure of the wave 1 emulator regression terms, for output CK-wt

	Estimate	Std. Error	t value	Pr(>|t|)
(Intercept)	-0.61	0.05	-13.17	0.00
k13	0.38	0.04	10.16	0.00
k2c	0.15	0.01	10.80	0.00
k2a	-0.55	0.04	-13.96	0.00
k6a	-0.07	0.01	-9.04	0.00
k2	0.21	0.03	7.75	0.00
k2b	-0.18	0.03	-5.74	0.00
k12a	-0.22	0.03	-7.57	0.00
k3	0.42	0.05	8.91	0.00
k5	0.15	0.02	5.92	0.00
k9	0.25	0.03	7.94	0.00
k1	0.26	0.03	7.45	0.00
k16a	-1.17	0.17	-6.91	0.00
k7	0.15	0.02	6.06	0.00
I(k13^2)	-0.29	0.04	-6.90	0.00
I(k2a^2)	-0.38	0.05	-7.85	0.00
I(k16a^2)	-0.80	0.16	-4.90	0.00
I(k2c^2)	0.03	0.01	5.16	0.00
I(k13^3)	0.08	0.02	3.18	0.00
k13:k2a	0.69	0.04	15.45	0.00
k13:k2c	-0.20	0.02	-11.58	0.00
k2a:I(k13^2)	-0.26	0.03	-8.08	0.00
k13:k2b	0.26	0.04	5.93	0.00
k13:k12a	0.31	0.04	7.49	0.00
k2c:k2a	0.21	0.02	9.89	0.00
k13:k2	-0.15	0.02	-6.23	0.00
k13:k6a	0.06	0.01	6.75	0.00
k2c:I(k13^2)	0.05	0.01	5.29	0.00
k13:k1	-0.14	0.02	-5.58	0.00
k2a:k12a	-0.25	0.04	-5.93	0.00
k13:I(k2a^2)	0.24	0.05	5.11	0.00
k2a:k2	0.17	0.04	4.69	0.00
k2:k1	-0.22	0.04	-5.24	0.00
k2c:k7	0.04	0.01	3.16	0.00
k2a:k2b	-0.26	0.04	-5.94	0.00
k3:k16a	0.30	0.07	4.11	0.00
k2a:k1	0.26	0.04	6.04	0.00
k3:k9	-0.18	0.04	-4.80	0.00
k3:I(k13^2)	-0.08	0.02	-3.93	0.00
k2b:k12a	-0.21	0.04	-4.97	0.00
k2b:k1	0.17	0.04	4.09	0.00
k13:k9	-0.11	0.02	-4.32	0.00
k2c:k2	-0.04	0.01	-3.12	0.00
k2:k2b	0.12	0.04	3.34	0.00
k13:k5	-0.08	0.02	-3.16	0.00
k2a:k3	0.14	0.04	3.66	0.00
k2a:k6a	-0.07	0.01	-4.79	0.00
k2a:I(k2c^2)	0.04	0.01	5.19	0.00
k13:I(k2c^2)	-0.02	0.01	-4.48	0.00
k9:I(k2c^2)	-0.01	0.00	-3.12	0.00
k2c:k2b	0.06	0.01	4.42	0.00
k12a:I(k13^2)	-0.10	0.03	-3.24	0.00
k2a:k9	0.11	0.04	2.92	0.00
k2b:I(k13^2)	-0.10	0.03	-2.99	0.00
k1:I(k16a^2)	-0.21	0.07	-2.96	0.00
k13:k2c:k2a	-0.08	0.01	-5.83	0.00
k13:k2a:k1	-0.19	0.04	-4.45	0.00
k13:k2:k1	0.12	0.04	2.97	0.00
k13:k2b:k1	-0.12	0.04	-2.81	0.00
k13:k2a:k6a	0.05	0.01	3.59	0.00
k2a:k2:k1	-0.23	0.06	-3.67	0.00
k13:k2a:k12a	0.16	0.04	3.73	0.00
k13:k2b:k12a	0.16	0.04	3.61	0.00
k13:k2c:k2b	-0.04	0.01	-3.00	0.00
k13:k2a:k2b	0.15	0.04	3.54	0.00
k2a:k3:k9	-0.22	0.07	-3.28	0.00

Table 4 The structure of the wave 1 emulator regression terms, for output Auxin-mu

	Estimate	Std. Error	t value	Pr(>|t|)
(Intercept)	0.88	0.03	27.97	0.00
k1vauxin	1.43	0.02	58.86	0.00
k2	-0.49	0.03	-15.31	0.00
k13	0.14	0.02	6.05	0.00
k17	-0.41	0.02	-19.18	0.00
k1	-0.31	0.02	-17.84	0.00
k2c	0.04	0.01	6.14	0.00
k2a	-0.16	0.02	-6.87	0.00
k19	-0.06	0.01	-5.79	0.00
k2b	-0.10	0.02	-5.10	0.00
k12a	-0.03	0.02	-1.50	0.13
k16a	-0.55	0.11	-5.14	0.00
I(k1vauxin^2)	0.51	0.03	19.65	0.00
I(k2^2)	-0.17	0.03	-5.89	0.00
I(k13^2)	-0.05	0.02	-3.58	0.00
I(k1vauxin^3)	-0.10	0.02	-6.60	0.00
I(k2a^2)	-0.13	0.03	-3.81	0.00
I(k16a^2)	-0.38	0.10	-3.66	0.00
k2:k17	0.38	0.03	12.43	0.00
k1vauxin:k2	-0.40	0.03	-15.72	0.00
k13:k2a	0.22	0.03	7.85	0.00
k2:k1	0.28	0.03	9.63	0.00
k1vauxin:k17	-0.24	0.03	-9.36	0.00
k1vauxin:k1	-0.18	0.02	-11.29	0.00
k1vauxin:k13	0.17	0.02	9.11	0.00
k13:k2c	-0.06	0.01	-6.70	0.00
k1vauxin:k2a	-0.20	0.02	-9.59	0.00
k2:k13	-0.07	0.02	-3.83	0.00
k1vauxin:I(k13^2)	-0.07	0.01	-5.23	0.00
k17:k1	0.15	0.02	6.41	0.00
k13:k2b	0.10	0.02	6.32	0.00
k2:I(k1vauxin^2)	0.12	0.02	6.08	0.00
k2:k2a	0.21	0.03	8.04	0.00
k1vauxin:I(k2^2)	-0.15	0.03	-4.82	0.00
k2c:k2a	0.06	0.01	5.67	0.00
k1:k2a	0.18	0.03	6.59	0.00
k13:k1	-0.08	0.02	-5.00	0.00
k2a:I(k13^2)	-0.10	0.02	-4.94	0.00
k1vauxin:k2c	0.04	0.01	6.64	0.00
k1:k2b	0.12	0.03	4.62	0.00
k13:k19	0.04	0.01	3.42	0.00
k1vauxin:I(k2a^2)	-0.14	0.03	-4.62	0.00
k2a:k2b	-0.08	0.02	-3.31	0.00
k1vauxin:k2b	-0.06	0.02	-3.78	0.00
k2:k19	0.08	0.02	4.45	0.00
k2:k2b	0.14	0.03	5.57	0.00
k2:k2c	-0.05	0.01	-5.66	0.00
k19:k2b	0.06	0.02	3.85	0.00
k13:k12a	0.03	0.02	1.70	0.09
k2:k16a	0.15	0.05	3.34	0.00
k2c:I(k13^2)	0.02	0.01	3.46	0.00
k19:k12a	0.07	0.02	4.12	0.00
k17:k2a	0.07	0.02	2.99	0.00
k17:k19	0.05	0.02	3.08	0.00
k17:I(k1vauxin^2)	0.06	0.02	2.79	0.01
k17:k2c	-0.02	0.01	-2.86	0.00
k1vauxin:k12a	-0.05	0.02	-2.96	0.00
k13:I(k2a^2)	0.09	0.03	3.06	0.00
k1vauxin:k13:k2a	0.16	0.02	8.97	0.00
k2:k17:k1	-0.27	0.04	-6.70	0.00
k13:k2c:k2a	-0.06	0.01	-7.01	0.00
k2:k13:k2a	-0.13	0.03	-4.86	0.00
k2:k13:k1	0.12	0.03	4.61	0.00
k13:k1:k2a	-0.12	0.03	-4.54	0.00
k1vauxin:k13:k2c	-0.03	0.01	-4.79	0.00
k1vauxin:k2c:k2a	0.04	0.01	4.32	0.00
k2:k1:k2a	-0.18	0.04	-4.50	0.00
k2:k13:k2c	0.04	0.01	4.20	0.00
k2:k1:k2b	-0.13	0.04	-3.32	0.00
k13:k19:k12a	-0.05	0.02	-3.03	0.00
k1vauxin:k2:k17	0.08	0.03	2.82	0.00
k1:k2a:k2b	0.12	0.04	3.03	0.00
k1vauxin:k2:k1	0.10	0.03	3.53	0.00
k2:k17:k2a	-0.11	0.04	-2.69	0.01
k2:k13:k2b	-0.09	0.03	-3.36	0.00
k2:k13:k19	-0.05	0.02	-2.82	0.00
k2:k17:k19	-0.08	0.03	-3.14	0.00
k13:k1:k2b	-0.08	0.03	-2.79	0.01

Table 5 The structure of the wave 1 emulator regression terms, for output Auxin-fa

	Estimate	Std. Error	t value	Pr(>|t|)
(Intercept)	0.58	0.04	16.31	0.00
k2c	-0.23	0.01	-19.62	0.00
k13	-0.45	0.03	-16.94	0.00
k6a	0.18	0.02	11.65	0.00
k1veth	0.57	0.04	13.51	0.00
k2a	0.60	0.03	18.40	0.00
k19	0.17	0.03	5.70	0.00
k18	-0.14	0.02	-7.89	0.00
k2	-0.09	0.03	-3.44	0.00
k9	-0.20	0.03	-8.03	0.00
k2b	0.13	0.02	6.84	0.00
k7	-0.22	0.02	-9.79	0.00
k1	-0.05	0.03	-1.62	0.11
k5	-0.04	0.02	-2.20	0.03
I(k13^2)	0.05	0.02	2.29	0.02
I(k1veth^2)	0.23	0.04	5.95	0.00
I(k2c^2)	-0.01	0.00	-2.90	0.00
I(k2a^2)	0.17	0.04	4.31	0.00
I(k6a^2)	-0.01	0.00	-3.33	0.00
I(k19^2)	-0.14	0.04	-3.57	0.00
I(k19^3)	-0.07	0.02	-2.97	0.00
k13:k1veth	-0.36	0.03	-11.23	0.00
k1veth:k2a	0.57	0.04	13.33	0.00
k2c:k1veth	-0.22	0.01	-15.07	0.00
k13:k2a	-0.22	0.03	-8.63	0.00
k2c:k13	0.09	0.01	12.12	0.00
k13:k6a	-0.11	0.01	-8.71	0.00
k13:k18	0.10	0.02	6.79	0.00
k1veth:k19	0.05	0.03	1.81	0.07
k6a:k1veth	0.11	0.01	8.47	0.00
k13:k7	0.14	0.02	6.18	0.00
k2c:k19	-0.05	0.01	-6.57	0.00
k19:I(k13^2)	-0.07	0.01	-5.98	0.00
k2c:k2a	-0.06	0.01	-5.56	0.00
k6a:k2a	0.07	0.01	5.37	0.00
k1veth:k7	-0.24	0.04	-6.18	0.00
k2a:k9	-0.19	0.04	-4.79	0.00
k1veth:k18	-0.09	0.03	-2.95	0.00
k1veth:k1	-0.15	0.03	-4.48	0.00
k2a:k7	-0.22	0.04	-5.50	0.00
k1veth:k9	-0.15	0.03	-4.37	0.00
k13:k9	0.07	0.02	3.32	0.00
k2a:k18	-0.06	0.03	-2.13	0.03
k2c:k2	0.05	0.01	4.08	0.00
k13:k2	0.08	0.02	3.54	0.00
k6a:I(k2c^2)	-0.01	0.00	-4.31	0.00
k6a:k2	-0.04	0.01	-3.66	0.00
k19:k18	0.05	0.02	3.53	0.00
k6a:I(k13^2)	0.03	0.01	3.29	0.00
k2a:k19	0.04	0.03	1.44	0.15
k1veth:I(k2a^2)	0.21	0.07	3.08	0.00
k1veth:k2b	0.11	0.03	3.25	0.00
k2c:k9	-0.03	0.01	-3.08	0.00
k1veth:k2	-0.05	0.04	-1.25	0.21
k2:k7	0.10	0.03	2.82	0.00
k1:I(k1veth^2)	-0.18	0.07	-2.77	0.01
k6a:I(k19^2)	-0.02	0.01	-2.74	0.01
k2c:k13:k1veth	0.09	0.01	7.21	0.00
k13:k6a:k1veth	-0.06	0.01	-4.41	0.00
k13:k1veth:k18	0.08	0.03	2.95	0.00
k13:k1veth:k2a	-0.16	0.04	-3.99	0.00
k2c:k1veth:k19	-0.05	0.01	-3.77	0.00
k13:k1veth:k7	0.15	0.04	3.91	0.00
k13:k2a:k7	0.16	0.04	3.99	0.00
k1veth:k19:k18	0.11	0.03	4.08	0.00
k2c:k2a:k9	-0.06	0.02	-3.23	0.00
k2c:k1veth:k2a	-0.06	0.02	-2.80	0.01
k2c:k1veth:k2	0.06	0.02	3.06	0.00
k13:k6a:k2a	-0.04	0.01	-2.97	0.00
k2a:k19:k18	0.08	0.03	2.96	0.00

Table 6 The structure of the wave 1 emulator regression terms, for output Auxin-fe

	Estimate	Std. Error	t value	Pr(>|t|)
(Intercept)	-0.07	0.01	-7.49	0.00
k11	0.09	0.01	13.60	0.00
k15	0.09	0.01	14.64	0.00
k13	-0.08	0.01	-12.75	0.00
k1veth	-0.01	0.01	-1.26	0.21
k17	0.04	0.01	3.56	0.00
k16a	-0.27	0.06	-4.25	0.00
k3a	-0.03	0.01	-4.12	0.00
k19	-0.00	0.01	-0.60	0.55
k2	0.01	0.00	3.52	0.00
k3	0.04	0.01	4.58	0.00
k12a	0.02	0.01	2.98	0.00
I(k16a^2)	-0.48	0.14	-3.50	0.00
I(k13^3)	0.01	0.00	3.65	0.00
I(k16a^3)	-0.30	0.09	-3.39	0.00
I(k11^3)	-0.00	0.00	-3.12	0.00
I(k19^2)	0.02	0.01	3.77	0.00
k15:k16a	0.19	0.03	7.02	0.00
k11:k16a	0.15	0.03	5.72	0.00
k13:k1veth	-0.07	0.01	-10.26	0.00
k13:k16a	-0.16	0.03	-5.82	0.00
k1veth:k16a	-0.12	0.04	-2.82	0.00
k17:k16a	0.15	0.04	3.47	0.00
k11:k1veth	0.06	0.01	8.00	0.00
k15:k1veth	0.05	0.01	7.13	0.00
k16a:k3a	-0.05	0.01	-4.02	0.00
k16a:k19	-0.00	0.01	-0.29	0.77
k11:k17	-0.04	0.01	-5.72	0.00
k11:k3a	0.02	0.00	5.52	0.00
k13:k3a	-0.04	0.01	-5.99	0.00
k16a:k12a	0.03	0.01	2.14	0.03
k15:k3a	0.01	0.00	3.96	0.00
k13:k17	0.03	0.01	4.61	0.00
k15:k3	-0.04	0.01	-5.24	0.00
k19:k3	0.03	0.01	4.65	0.00
k15:k17	-0.03	0.01	-5.02	0.00
k13:k12a	0.04	0.01	5.63	0.00
k16a:k3	0.05	0.01	4.06	0.00
k1veth:k17	0.04	0.01	3.07	0.00
k15:I(k16a^2)	0.11	0.03	4.43	0.00
k1veth:k3a	-0.01	0.01	-1.06	0.29
k11:I(k16a^2)	0.09	0.03	3.30	0.00
k1veth:k2	0.01	0.01	1.43	0.15
k11:k19	0.01	0.00	2.98	0.00
k13:I(k16a^2)	-0.10	0.03	-3.75	0.00
k11:k3	-0.02	0.00	-4.35	0.00
k1veth:I(k16a^2)	-0.13	0.04	-3.21	0.00
k17:I(k16a^2)	0.13	0.04	3.20	0.00
k11:k2	-0.01	0.00	-3.26	0.00
k15:I(k19^2)	-0.01	0.00	-3.67	0.00
k16a:I(k19^2)	0.03	0.01	3.65	0.00
k13:k1veth:k16a	-0.08	0.01	-6.55	0.00
k15:k1veth:k16a	0.05	0.01	4.15	0.00
k11:k1veth:k16a	0.06	0.01	5.15	0.00
k13:k16a:k12a	0.05	0.01	4.17	0.00
k16a:k19:k3	0.05	0.01	4.42	0.00
k1veth:k17:k16a	0.06	0.02	3.27	0.00
k13:k16a:k3a	-0.05	0.01	-3.89	0.00
k15:k16a:k3	-0.05	0.01	-3.86	0.00
k15:k17:k16a	-0.04	0.01	-3.28	0.00
k13:k1veth:k3a	-0.02	0.01	-3.73	0.00
k13:k17:k16a	0.04	0.01	2.99	0.00
k11:k19:k3	-0.01	0.00	-3.08	0.00
k11:k17:k16a	-0.04	0.01	-3.49	0.00
k11:k1veth:k17	-0.02	0.01	-3.18	0.00
k13:k1veth:k17	0.02	0.01	3.05	0.00
k11:k1veth:k2	-0.02	0.01	-2.75	0.01

Table 7 The structure of the wave 1 emulator regression terms, for output Auxin-mufe

	Estimate	Std. Error	t value	Pr(>|t|)
(Intercept)	0.01	0.02	0.90	0.37
k18	0.12	0.02	7.77	0.00
k1vauxin	0.06	0.01	4.66	0.00
k19	-0.13	0.02	-8.00	0.00
k12a	0.12	0.02	7.18	0.00
k2	-0.01	0.01	-0.93	0.35
k17	-0.04	0.02	-2.32	0.02
k3	0.01	0.01	0.74	0.46
k1	-0.04	0.01	-2.70	0.01
k10a	-0.00	0.01	-0.36	0.72
k9	-0.02	0.01	-1.15	0.25
I(k18^2)	0.03	0.01	2.78	0.01
I(k2^2)	0.03	0.03	1.17	0.24
I(k19^2)	0.09	0.02	4.52	0.00
I(k19^3)	0.04	0.01	3.58	0.00
k18:k1vauxin	0.09	0.01	6.55	0.00
k18:k19	-0.12	0.01	-12.82	0.00
k1vauxin:k19	-0.09	0.01	-9.86	0.00
k18:k12a	0.09	0.01	6.88	0.00
k1vauxin:k12a	0.08	0.01	6.31	0.00
k19:k17	-0.01	0.02	-0.41	0.68
k1vauxin:I(k18^2)	0.11	0.01	10.39	0.00
k19:k2	0.08	0.01	6.47	0.00
k1vauxin:k17	-0.02	0.02	-1.32	0.19
k12a:k17	-0.04	0.02	-1.60	0.11
k19:k1	0.07	0.01	5.77	0.00
k1vauxin:k3	0.03	0.01	2.32	0.02
k19:k3	-0.04	0.01	-3.23	0.00
k12a:k1	-0.08	0.02	-4.42	0.00
k18:k2	-0.03	0.02	-1.97	0.05
k12a:k2	-0.05	0.02	-2.20	0.03
k1vauxin:I(k2^2)	-0.11	0.02	-5.01	0.00
k18:k17	-0.05	0.02	-2.47	0.01
k18:I(k2^2)	-0.08	0.02	-3.54	0.00
k19:k12a	-0.13	0.02	-6.73	0.00
k19:k10a	0.04	0.01	3.68	0.00
k9:I(k18^2)	0.04	0.01	4.04	0.00
k12a:k3	0.07	0.02	4.10	0.00
k18:k1	-0.04	0.01	-3.76	0.00
k17:k3	0.04	0.02	1.79	0.07
k1vauxin:k2	-0.03	0.01	-2.06	0.04
k2:k1	0.01	0.02	0.65	0.52
k2:k17	0.02	0.02	0.96	0.34
k17:I(k18^2)	0.06	0.02	4.02	0.00
k12a:k10a	-0.06	0.02	-3.47	0.00
k12a:I(k19^2)	-0.08	0.01	-5.38	0.00
k17:I(k19^2)	-0.06	0.01	-3.82	0.00
k19:I(k2^2)	0.07	0.02	2.97	0.00
k18:k1vauxin:k19	-0.14	0.01	-15.57	0.00
k18:k1vauxin:k12a	0.12	0.01	9.06	0.00
k1vauxin:k19:k17	0.09	0.01	6.69	0.00
k1vauxin:k19:k3	-0.07	0.01	-5.57	0.00
k19:k12a:k17	0.13	0.02	6.66	0.00
k18:k19:k17	0.07	0.01	5.15	0.00
k18:k1vauxin:k17	-0.05	0.01	-3.94	0.00
k1vauxin:k12a:k17	-0.09	0.02	-4.24	0.00
k1vauxin:k17:k3	0.07	0.02	3.34	0.00
k19:k2:k1	-0.08	0.02	-4.06	0.00
k18:k19:k2	0.04	0.01	3.32	0.00
k19:k2:k17	-0.09	0.02	-4.49	0.00
k18:k1vauxin:k2	-0.04	0.01	-3.23	0.00
k1vauxin:k12a:k2	-0.06	0.02	-2.91	0.00

Table 8 The structure of the wave 1 emulator regression terms, for output ET-fa

	Estimate	Std. Error	t value	Pr(>|t|)
(Intercept)	2.28	0.01	187.75	0.00
k1veth	2.03	0.01	140.83	0.00
k19	0.22	0.01	21.09	0.00
k12a	-0.21	0.01	-21.64	0.00
k18	-0.18	0.01	-17.74	0.00
k17	-0.07	0.01	-6.69	0.00
k3	0.06	0.01	5.55	0.00
k2	-0.04	0.01	-3.70	0.00
k3a	0.03	0.01	3.31	0.00
k1	-0.02	0.01	-2.28	0.02
I(k1veth^2)	0.30	0.02	17.65	0.00
I(k19^2)	-0.16	0.01	-23.97	0.00
I(k12a^2)	-0.14	0.01	-9.47	0.00
I(k18^3)	0.03	0.00	7.01	0.00
I(k3a^2)	0.05	0.01	3.16	0.00
I(k3^2)	0.05	0.01	3.43	0.00
k19:k12a	0.33	0.01	36.66	0.00
k1veth:k19	0.06	0.02	4.20	0.00
k19:k18	0.13	0.01	22.86	0.00
k1veth:k12a	-0.08	0.02	-4.81	0.00
k18:k17	-0.12	0.01	-11.72	0.00
k19:k17	0.10	0.01	10.38	0.00
k12a:k18	-0.14	0.01	-16.20	0.00
k1veth:k18	-0.06	0.01	-6.27	0.00
k18:k3	0.09	0.01	9.04	0.00
k19:k3	-0.07	0.01	-6.97	0.00
k19:k3a	-0.07	0.01	-7.46	0.00
k12a:k17	-0.10	0.02	-6.34	0.00
k19:k2	0.07	0.01	6.92	0.00
k1veth:k17	-0.01	0.02	-0.79	0.43
k1veth:I(k19^2)	-0.11	0.01	-9.32	0.00
k18:k3a	0.07	0.01	6.85	0.00
k3:k3a	-0.01	0.02	-0.85	0.40
k17:k3	0.04	0.02	2.42	0.02
k12a:k2	-0.09	0.01	-6.96	0.00
k18:k2	-0.05	0.01	-5.08	0.00
k1veth:k3	0.00	0.02	0.01	0.99
k12a:k3	0.06	0.02	3.71	0.00
k17:k3a	-0.01	0.02	-0.63	0.53
k1veth:k3a	0.05	0.02	2.96	0.00
k12a:k3a	0.08	0.01	6.39	0.00
k19:k1	0.05	0.01	5.58	0.00
k19:I(k1veth^2)	-0.09	0.02	-5.13	0.00
k12a:k1	-0.06	0.01	-4.71	0.00
k1veth:k2	-0.02	0.02	-1.40	0.16
k18:k1	-0.04	0.01	-3.98	0.00
k1veth:I(k12a^2)	-0.11	0.03	-4.43	0.00
k2:k1	0.02	0.02	1.12	0.26
k17:k2	0.00	0.02	0.05	0.96
k1veth:k1	-0.04	0.01	-3.06	0.00
k1veth:k19:k12a	0.22	0.02	14.36	0.00
k1veth:k19:k18	0.11	0.01	10.76	0.00
k1veth:k18:k17	-0.12	0.02	-7.99	0.00
k1veth:k12a:k18	-0.13	0.02	-8.79	0.00
k1veth:k12a:k17	-0.18	0.02	-7.78	0.00
k19:k18:k3	-0.08	0.01	-7.38	0.00
k12a:k18:k17	-0.10	0.01	-6.40	0.00
k1veth:k19:k17	0.11	0.02	6.90	0.00
k19:k18:k17	0.06	0.01	5.50	0.00
k1veth:k19:k3	-0.10	0.02	-6.52	0.00
k19:k17:k3a	0.10	0.02	6.47	0.00
k18:k3:k3a	-0.09	0.02	-5.56	0.00
k18:k17:k3	0.09	0.02	5.71	0.00
k12a:k17:k3a	-0.10	0.02	-4.36	0.00
k19:k3:k3a	0.07	0.02	4.65	0.00
k1veth:k19:k3a	-0.07	0.02	-4.51	0.00
k1veth:k18:k3	0.05	0.02	3.35	0.00
k1veth:k19:k2	0.06	0.02	3.72	0.00
k19:k18:k3a	-0.03	0.01	-3.25	0.00
k1veth:k3:k3a	-0.08	0.02	-3.59	0.00
k17:k3:k3a	0.07	0.02	3.22	0.00
k19:k17:k2	-0.09	0.02	-6.01	0.00
k12a:k17:k2	0.09	0.02	3.87	0.00
k19:k2:k1	-0.05	0.02	-3.25	0.00
k19:k18:k2	0.03	0.01	3.18	0.00
k1veth:k12a:k1	-0.07	0.02	-3.08	0.00
k12a:k18:k3	0.04	0.01	2.95	0.00
k18:k17:k3a	-0.04	0.02	-2.77	0.01

Table 9 The structure of the wave 1 emulator regression terms, for output ET-fe

	Estimate	Std. Error	t value	Pr(>|t|)
(Intercept)	-0.69	0.02	-29.00	0.00
k1vauxin	-1.05	0.02	-45.52	0.00
k18	0.41	0.02	19.23	0.00
k3	0.21	0.02	10.71	0.00
k13	-0.12	0.01	-8.24	0.00
k2	0.23	0.01	15.31	0.00
k3a	0.10	0.01	7.20	0.00
k1	0.13	0.01	8.59	0.00
k2a	0.14	0.02	7.97	0.00
k2b	0.06	0.01	4.52	0.00
k17	0.05	0.02	2.48	0.01
I(k1vauxin^2)	-0.43	0.01	-37.15	0.00
I(k3^2)	0.11	0.03	3.86	0.00
I(k13^2)	0.03	0.01	2.59	0.01
I(k18^2)	0.10	0.02	4.84	0.00
I(k2^2)	0.14	0.03	5.08	0.00
I(k18^3)	-0.07	0.01	-5.77	0.00
I(k17^2)	0.06	0.03	2.24	0.03
I(k3a^2)	0.07	0.02	3.30	0.00
k1vauxin:k18	0.29	0.02	16.04	0.00
k1vauxin:k3	0.11	0.02	6.35	0.00
k18:k2	-0.14	0.02	-9.35	0.00
k3a:k17	-0.24	0.02	-12.38	0.00
k18:k17	-0.26	0.02	-12.62	0.00
k1vauxin:k3a	0.14	0.01	10.53	0.00
k13:k2a	-0.12	0.02	-6.92	0.00
k1vauxin:k2	0.18	0.01	12.43	0.00
k18:k1	-0.09	0.01	-6.25	0.00
k1vauxin:k13	-0.13	0.01	-8.88	0.00
k17:I(k1vauxin^2)	-0.07	0.01	-7.03	0.00
k3:k3a	-0.07	0.02	-3.37	0.00
k18:k3	0.14	0.02	6.20	0.00
k18:k2a	-0.09	0.02	-5.04	0.00
k18:k13	0.06	0.01	7.08	0.00
k1vauxin:k1	0.10	0.01	6.61	0.00
k1vauxin:k2a	0.15	0.02	8.75	0.00
k18:I(k1vauxin^2)	0.06	0.01	5.51	0.00
k1vauxin:I(k18^2)	0.09	0.01	8.29	0.00
k17:I(k18^2)	0.10	0.02	6.02	0.00
k3:k13	0.05	0.02	3.12	0.00
k1vauxin:I(k3^2)	0.16	0.03	6.08	0.00
k3:I(k18^2)	-0.11	0.02	-6.47	0.00
k3:k2	-0.11	0.02	-5.47	0.00
k1vauxin:I(k2^2)	0.13	0.03	5.13	0.00
k2:k2a	-0.05	0.02	-2.83	0.00
k2:k3a	-0.10	0.02	-5.30	0.00
k18:I(k2^2)	-0.12	0.02	-4.62	0.00
k13:k2b	-0.06	0.01	-4.65	0.00
k3a:k2a	-0.09	0.03	-3.76	0.00
k13:k3a	0.05	0.01	4.22	0.00
k1vauxin:I(k13^2)	0.05	0.01	4.27	0.00
k1vauxin:I(k17^2)	0.11	0.03	4.16	0.00
k3:k1	-0.08	0.02	-4.07	0.00
k18:I(k3^2)	-0.08	0.03	-3.01	0.00
k3:k2a	-0.06	0.02	-2.99	0.00
k1:k2b	-0.07	0.02	-3.49	0.00
k1:k2a	-0.06	0.02	-3.22	0.00
k1:k17	0.06	0.02	3.04	0.00
k2:k1	-0.09	0.02	-4.35	0.00
k2a:k2b	0.05	0.02	2.69	0.01
k1vauxin:k18:k3	0.11	0.02	7.18	0.00
k1vauxin:k18:k2	-0.11	0.02	-6.85	0.00
k18:k13:k2a	0.08	0.02	5.41	0.00
k1vauxin:k13:k2a	-0.07	0.02	-4.95	0.00
k1vauxin:k3:k3a	-0.11	0.02	-4.96	0.00
k1vauxin:k3:k13	0.07	0.01	4.69	0.00
k13:k3a:k2a	0.09	0.02	4.13	0.00
k3:k3a:k2a	0.11	0.03	3.33	0.00
k1vauxin:k3a:k2a	-0.07	0.02	-3.11	0.00
k1vauxin:k18:k2a	-0.05	0.02	-3.38	0.00
k1vauxin:k18:k1	-0.05	0.02	-3.04	0.00
k18:k2:k1	0.08	0.02	3.55	0.00
k1:k2a:k2b	-0.11	0.03	-3.16	0.00

Table 10 The structure of the wave 1 emulator regression terms, for output CK-fa

	Estimate	Std. Error	t value	Pr(>|t|)
(Intercept)	-0.46	0.03	-16.01	0.00
k13	0.45	0.02	20.67	0.00
k2c	0.17	0.01	17.86	0.00
k18	0.19	0.02	10.08	0.00
k6a	-0.11	0.01	-8.76	0.00
k1veth	-0.50	0.04	-13.15	0.00
k2a	-0.46	0.03	-15.72	0.00
k19	-0.09	0.02	-4.19	0.00
k2b	-0.07	0.02	-4.87	0.00
k9	0.13	0.02	7.49	0.00
k3	0.14	0.02	7.62	0.00
k3a	0.18	0.02	8.79	0.00
I(k13^2)	-0.10	0.02	-6.72	0.00
I(k1veth^2)	-0.25	0.03	-7.10	0.00
I(k6a^2)	0.01	0.00	4.17	0.00
I(k2a^2)	-0.10	0.03	-2.76	0.01
I(k2c^2)	0.01	0.00	2.92	0.00
I(k19^2)	0.13	0.03	4.35	0.00
k13:k1veth	0.39	0.03	11.79	0.00
k13:k2a	0.23	0.02	11.51	0.00
k1veth:k2a	-0.56	0.04	-14.84	0.00
k13:k18	-0.15	0.01	-11.31	0.00
k18:k1veth	0.24	0.03	9.41	0.00
k18:k2a	0.21	0.02	11.64	0.00
k13:k2c	-0.07	0.01	-11.76	0.00
k2c:k1veth	0.21	0.02	12.21	0.00
k13:k6a	0.10	0.01	9.78	0.00
k2c:k18	-0.05	0.01	-7.72	0.00
k18:k6a	0.05	0.01	7.38	0.00
k2c:k2a	0.05	0.01	5.80	0.00
k13:k19	0.07	0.01	5.60	0.00
k1veth:k19	-0.05	0.02	-2.50	0.01
k2c:k19	0.03	0.01	5.00	0.00
k2a:k9	0.15	0.03	4.89	0.00
k2a:I(k1veth^2)	-0.21	0.05	-4.07	0.00
k13:k3	-0.06	0.02	-3.43	0.00
k13:k9	-0.07	0.02	-4.11	0.00
k1veth:k9	0.12	0.03	4.40	0.00
k3:k3a	-0.09	0.03	-3.49	0.00
k13:k3a	-0.07	0.02	-4.14	0.00
k1veth:k3a	0.12	0.03	4.31	0.00
k6a:k1veth	-0.08	0.01	-6.64	0.00
k6a:k2a	-0.06	0.01	-5.44	0.00
k1veth:k3	0.11	0.03	3.97	0.00
k6a:I(k13^2)	-0.03	0.01	-3.75	0.00
k1veth:I(k13^2)	-0.08	0.02	-3.57	0.00
k2a:k3	0.10	0.03	3.74	0.00
k6a:I(k2c^2)	0.00	0.00	3.83	0.00
k19:k3a	0.06	0.02	3.27	0.00
k19:I(k19^2)	0.06	0.02	3.27	0.00
k18:I(k1veth^2)	0.11	0.03	3.29	0.00
k6a:I(k2a^2)	-0.05	0.02	-3.15	0.00
k1veth:I(k2a^2)	-0.15	0.05	-2.79	0.01
k1veth:I(k2c^2)	0.02	0.01	2.72	0.01
k13:k2c:k1veth	-0.07	0.01	-7.25	0.00
k13:k18:k1veth	-0.17	0.02	-8.20	0.00
k13:k1veth:k2a	0.21	0.03	6.88	0.00
k13:k18:k6a	-0.04	0.01	-5.32	0.00
k18:k1veth:k2a	0.18	0.03	5.61	0.00
k2c:k18:k1veth	-0.05	0.01	-5.25	0.00
k2c:k1veth:k2a	0.06	0.02	3.57	0.00
k2c:k1veth:k19	0.04	0.01	3.96	0.00
k13:k6a:k1veth	0.05	0.01	4.44	0.00
k13:k6a:k2a	0.04	0.01	4.02	0.00
k13:k2a:k9	-0.12	0.03	-3.76	0.00
k18:k6a:k1veth	0.03	0.01	2.76	0.01

Table 11 The structure of the wave 1 emulator regression terms, for output CK-fe

	Estimate	Std. Error	t value	Pr(>|t|)
(Intercept)	1.88	0.05	41.09	0.00
k18	-1.17	0.04	-32.32	0.00
k1vCK	1.76	0.04	46.12	0.00
k3	-1.04	0.05	-22.12	0.00
k17	0.65	0.03	21.33	0.00
k13	-0.24	0.02	-11.43	0.00
k3a	-0.43	0.02	-18.57	0.00
k2	0.33	0.02	17.54	0.00
k2a	0.33	0.02	15.25	0.00
k1	0.20	0.02	12.66	0.00
k16a	1.85	0.29	6.39	0.00
I(k18ˆ2)	0.40	0.03	15.53	0.00
I(k1vCKˆ2)	0.38	0.03	12.15	0.00
I(k13ˆ2)	0.18	0.03	6.86	0.00
I(k2ˆ2)	0.25	0.03	8.28	0.00
I(k16aˆ2)	2.83	0.66	4.32	0.00
I(k3aˆ2)	-0.11	0.03	-4.00	0.00
I(k2aˆ2)	0.14	0.03	4.37	0.00
I(k3ˆ2)	0.07	0.03	2.48	0.01
I(k18ˆ3)	-0.05	0.02	-3.12	0.00
I(k1vCKˆ3)	-0.14	0.05	-2.72	0.01
I(k13ˆ3)	-0.05	0.02	-2.93	0.00
k18:k3	0.61	0.04	15.81	0.00
k18:k17	-0.44	0.03	-16.83	0.00
k18:k1vCK	-0.27	0.02	-16.88	0.00
k3:k17	-0.62	0.03	-21.10	0.00
k18:k3a	0.23	0.02	14.34	0.00
k13:k2a	-0.42	0.03	-15.14	0.00
k3:k3a	0.52	0.03	18.03	0.00
k18:k2	-0.33	0.03	-12.54	0.00
k18:k1	-0.14	0.02	-8.82	0.00
k1vCK:k17	0.26	0.03	9.18	0.00
k1vCK:k3	-0.20	0.02	-8.06	0.00
k18:k2a	-0.14	0.02	-7.49	0.00
k18:k13	0.06	0.01	5.39	0.00
k3:k13	0.09	0.02	5.69	0.00
k13:k3a	0.10	0.02	6.44	0.00
k2a:I(k13ˆ2)	0.12	0.02	5.76	0.00
k2:I(k18ˆ2)	0.10	0.02	4.92	0.00
k3:k2	-0.14	0.02	-5.72	0.00
k18:k16a	-0.61	0.13	-4.72	0.00
k3:k16a	-0.86	0.19	-4.42	0.00
k1vCK:k3a	-0.12	0.02	-4.94	0.00
k1vCK:k2	0.12	0.02	4.87	0.00
k3a:k2a	-0.19	0.03	-6.68	0.00
k1vCK:k1	0.10	0.02	4.22	0.00
k3:k1	-0.11	0.02	-4.62	0.00
k18:I(k2ˆ2)	-0.15	0.03	-4.74	0.00
k3:k2a	-0.17	0.03	-5.98	0.00
k3:I(k18ˆ2)	-0.08	0.02	-4.05	0.00
k18:I(k16aˆ2)	-0.45	0.12	-3.59	0.00
k16a:I(k16aˆ2)	1.40	0.43	3.27	0.00
k17:I(k18ˆ2)	0.08	0.02	3.63	0.00
k17:I(k3aˆ2)	-0.16	0.05	-3.32	0.00
k3a:k2	-0.09	0.02	-3.69	0.00
k13:I(k2aˆ2)	-0.10	0.03	-3.15	0.00
k17:k13	-0.05	0.02	-3.37	0.00
k3a:I(k3ˆ2)	-0.16	0.05	-3.45	0.00
k17:I(k3ˆ2)	0.17	0.05	3.50	0.00
k1vCK:I(k3aˆ2)	-0.13	0.05	-2.75	0.01
k18:I(k1vCKˆ2)	0.09	0.03	2.76	0.01
k3:I(k16aˆ2)	-0.51	0.18	-2.76	0.01
k18:k3:k3a	-0.27	0.03	-9.63	0.00
k18:k3:k17	0.29	0.03	10.27	0.00
k18:k13:k2a	0.12	0.02	6.26	0.00
k13:k3a:k2a	0.12	0.03	4.24	0.00
k18:k1vCK:k17	-0.12	0.03	-4.05	0.00
k18:k3:k16a	0.20	0.05	3.71	0.00
k3:k13:k2a	0.12	0.03	4.39	0.00
k3:k3a:k2a	0.13	0.04	3.12	0.00

Table 12 The structure of the wave 1 emulator regression terms, for output CK-fc

	Estimate	Std. Error	t value	Pr(>|t|)
(Intercept)	0.69	0.03	19.90	0.00
k1vauxin	0.68	0.03	21.22	0.00
k5	0.44	0.03	14.78	0.00
k17	-0.46	0.02	-21.46	0.00
k2	-0.43	0.02	-17.82	0.00
k6a	-0.01	0.01	-0.60	0.55
k13	0.15	0.02	7.61	0.00
k3	0.24	0.02	12.64	0.00
k2c	0.05	0.01	8.44	0.00
k1	-0.29	0.03	-10.38	0.00
k3a	0.18	0.02	9.50	0.00
k18	-0.04	0.03	-1.31	0.19
k2a	-0.18	0.02	-8.10	0.00
k12a	-0.09	0.02	-3.77	0.00
I(k1vauxin^3)	-0.11	0.02	-6.14	0.00
I(k17^2)	0.11	0.04	2.81	0.00
I(k18^2)	-0.07	0.02	-3.70	0.00
I(k13^2)	-0.06	0.01	-4.11	0.00
I(k3a^2)	0.05	0.04	1.32	0.19
I(k3^2)	0.05	0.04	1.20	0.23
I(k1vauxin^2)	0.15	0.03	4.44	0.00
I(k2^2)	-0.10	0.03	-3.25	0.00
k1vauxin:k5	0.50	0.03	15.41	0.00
k1vauxin:k17	-0.32	0.02	-16.29	0.00
k17:k2	0.48	0.03	16.69	0.00
k1vauxin:k3	0.32	0.02	16.40	0.00
k1vauxin:k3a	0.19	0.02	9.94	0.00
k2:k1	0.24	0.03	7.43	0.00
k1vauxin:k2	-0.28	0.03	-9.06	0.00
k6a:k18	0.00	0.01	0.45	0.65
k17:k3a	-0.16	0.03	-4.72	0.00
k3:k3a	-0.19	0.03	-5.92	0.00
k2:k13	-0.15	0.02	-7.88	0.00
k13:k2a	0.12	0.02	5.19	0.00
k1vauxin:k13	0.10	0.01	7.76	0.00
k1vauxin:k18	-0.04	0.03	-1.62	0.11
k1vauxin:k6a	0.02	0.01	2.48	0.01
k5:k1	-0.11	0.03	-3.49	0.00
k5:k2	-0.18	0.03	-6.41	0.00
k6a:k12a	0.03	0.01	2.85	0.00
k17:k3	0.17	0.03	5.81	0.00
k17:k1	0.16	0.03	5.69	0.00
k1vauxin:k1	-0.10	0.02	-5.38	0.00
k13:k2c	-0.03	0.01	-5.03	0.00
k1vauxin:I(k18^2)	-0.07	0.02	-4.30	0.00
k1vauxin:k12a	-0.08	0.02	-3.29	0.00
k6a:I(k18^2)	0.03	0.01	3.90	0.00
k1vauxin:I(k17^2)	0.15	0.04	4.01	0.00
k2:k3a	-0.12	0.03	-4.30	0.00
k2:k2a	0.18	0.03	5.63	0.00
k13:k1	-0.08	0.02	-4.34	0.00
k1vauxin:I(k3a^2)	0.13	0.04	3.58	0.00
k5:k17	-0.02	0.03	-0.53	0.60
k18:k12a	-0.03	0.02	-1.28	0.20
k18:I(k3^2)	0.13	0.04	3.39	0.00
k1:I(k17^2)	0.20	0.06	3.70	0.00
k17:k2a	0.11	0.03	3.90	0.00
k2:k3	-0.06	0.03	-1.74	0.08
k1vauxin:k2a	-0.10	0.02	-4.31	0.00
k2:I(k1vauxin^2)	0.10	0.02	4.23	0.00
k18:I(k1vauxin^2)	-0.07	0.02	-3.86	0.00
k5:I(k2^2)	-0.26	0.06	-4.73	0.00
k1:k2a	0.09	0.03	3.12	0.00
k17:k2c	-0.03	0.01	-2.98	0.00
k5:I(k1vauxin^2)	0.07	0.03	2.84	0.00
k3:k1	-0.08	0.03	-2.87	0.00
k1vauxin:k17:k3a	-0.25	0.03	-7.53	0.00
k1vauxin:k6a:k18	0.06	0.01	8.11	0.00
k17:k2:k1	-0.34	0.05	-6.85	0.00
k1vauxin:k3:k3a	-0.17	0.03	-5.10	0.00
k17:k3:k3a	0.12	0.05	2.38	0.02
k1vauxin:k5:k17	-0.18	0.03	-5.41	0.00
k2:k13:k1	0.13	0.03	3.94	0.00
k1vauxin:k18:k12a	-0.08	0.02	-3.47	0.00
k1vauxin:k6a:k12a	0.04	0.01	3.89	0.00
k5:k17:k2	0.13	0.05	2.72	0.01
k1vauxin:k5:k1	-0.11	0.03	-3.26	0.00
k2:k1:k2a	-0.21	0.05	-4.47	0.00
k2:k13:k2a	-0.12	0.03	-3.62	0.00
k1vauxin:k13:k2a	0.07	0.02	3.03	0.00
k1vauxin:k2:k3	-0.10	0.03	-2.90	0.00

Table 13 The structure of the wave 1 emulator regression terms, for output PLSp-fa

	Estimate	Std. Error	t value	Pr(>|t|)
(Intercept)	-0.91	0.02	-36.44	0.00
k6a	0.64	0.02	38.05	0.00
k2c	-0.09	0.01	-10.94	0.00
k1veth	-1.03	0.03	-31.73	0.00
k13	0.29	0.02	15.49	0.00
k2a	0.15	0.03	5.41	0.00
k5	0.19	0.03	6.44	0.00
k2b	0.10	0.02	4.37	0.00
k7	-0.09	0.02	-4.24	0.00
k12a	0.22	0.03	8.46	0.00
I(k6a^3)	-0.02	0.00	-15.38	0.00
I(k1veth^2)	-0.30	0.05	-6.10	0.00
k6a:k1veth	0.45	0.02	29.50	0.00
k6a:k13	-0.14	0.01	-16.32	0.00
k1veth:k13	0.21	0.03	7.24	0.00
k13:k5	-0.17	0.03	-6.64	0.00
k6a:k12a	-0.07	0.01	-5.58	0.00
k2c:k1veth	-0.08	0.01	-6.31	0.00
k1veth:k2a	0.20	0.04	5.22	0.00
k6a:I(k1veth^2)	0.12	0.02	4.69	0.00
k1veth:k5	0.23	0.04	5.11	0.00
k2c:k13	0.03	0.01	3.59	0.00
k2a:k5	0.14	0.04	3.51	0.00
k5:k7	-0.13	0.04	-3.31	0.00
k1veth:k12a	0.13	0.04	3.26	0.00
k6a:k2a	0.04	0.01	3.16	0.00
k2c:k2a	-0.04	0.01	-3.12	0.00
k2c:k5	-0.04	0.01	-3.02	0.00
k6a:k1veth:k13	-0.07	0.01	-4.84	0.00
k1veth:k13:k5	-0.19	0.04	-4.27	0.00

Table 14 The structure of the wave 1 emulator regression terms, for output PLSp-fe

Parameter	Unit	Parameter	Unit
k_1	μM	k_{10a}	$\mu M^{-1}\ s^{-1}$
k_{1a}	$\mu M\ s^{-1}$	k_{11}	$\mu M^{-1}\ s^{-1}$
k_2	$\mu M\ s^{-1}$	k_{12}	$\mu M\ s^{-1}$
k_{2a}	s^{-1}	k_{12a}	$\mu M^{-1}\ s^{-1}$
k_{2b}	μM	k_{13}	s^{-1}
k_{2c}	μM	k_{14}	$\mu M^{-1}\ s^{-1}$
k_3	s^{-1}	k_{15}	s^{-1}
k_{3a}	$\mu M^{-1}\ s^{-1}$	k_{16}	$\mu M\ s^{-1}$
k_4	$\mu M^{-1}\ s^{-1}$	k_{16a}	s^{-1}
k_5	s^{-1}	k_{17}	s^{-1}
k_6	s^{-1}	k_{18}	μM
k_{6a}	μM	k_{18a}	$\mu M\ s^{-1}$
k_7	s^{-1}	k_{19}	s^{-1}
k_8	s^{-1}	$k_{1vauxin}$	s^{-1}
k_9	s^{-1}	k_{1vCK}	s^{-1}
k_{10}	s^{-1}	k_{1veth}	s^{-1}

Table 15 The dimensions or units of each of the rate constants that feature in Table 1 of the main article.

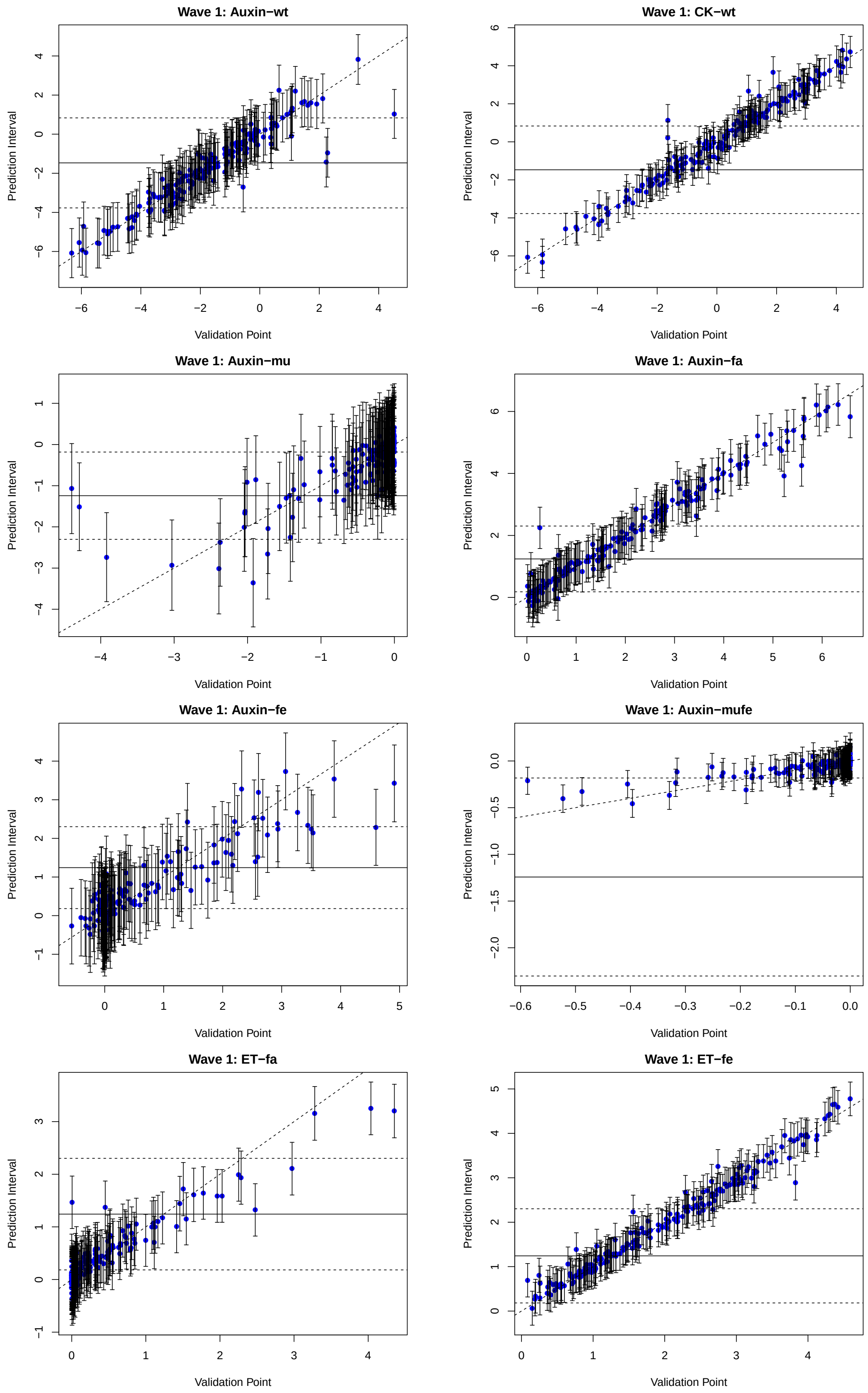

Table 16 Wave 1 emulator diagnostics for 8 out of 13 outputs considered. The horizontal lines give the target interval.

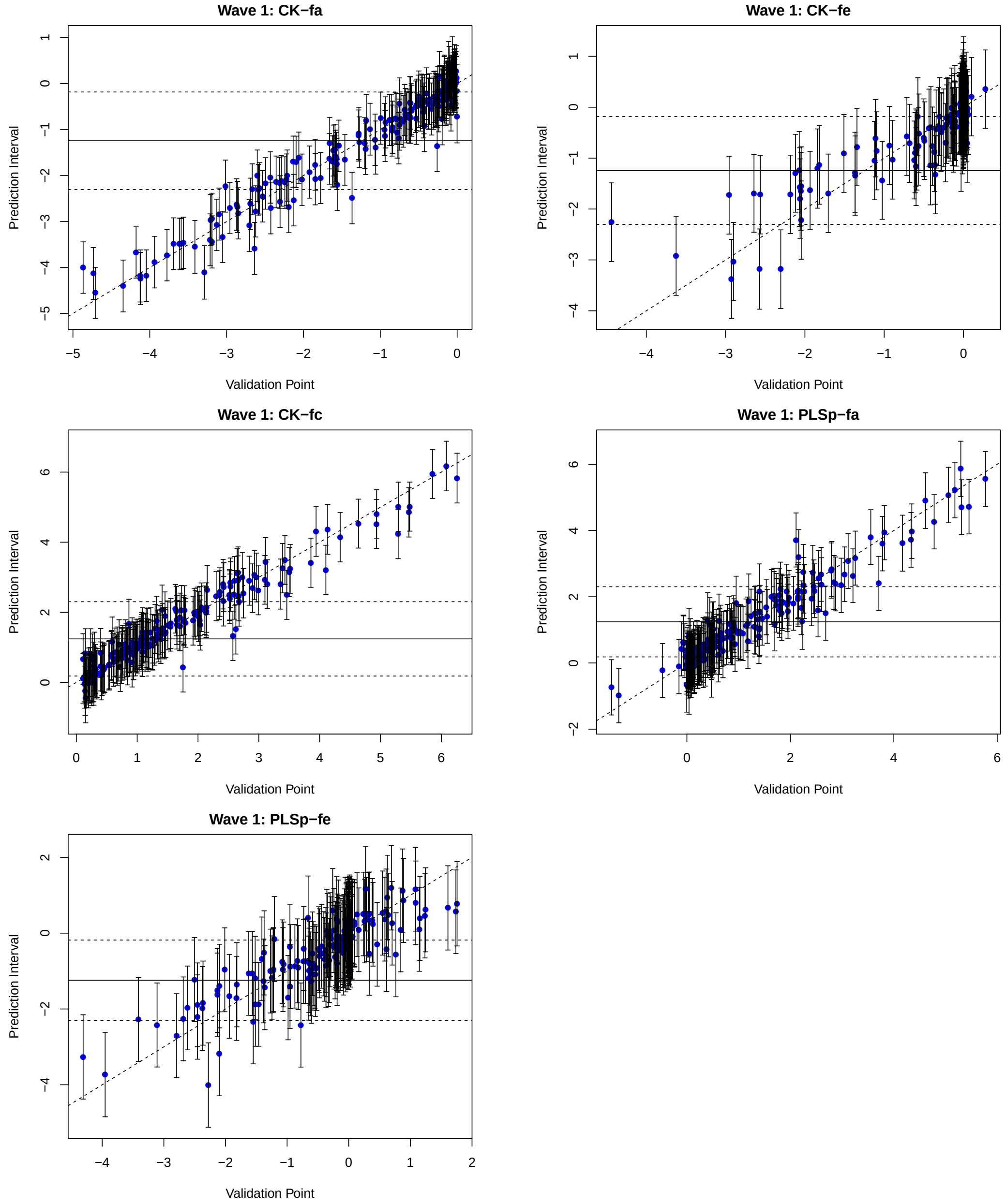

Table 17 Wave 1 emulator diagnostics for the remaining 5 out of 13 outputs considered. The horizontal lines give the target interval.

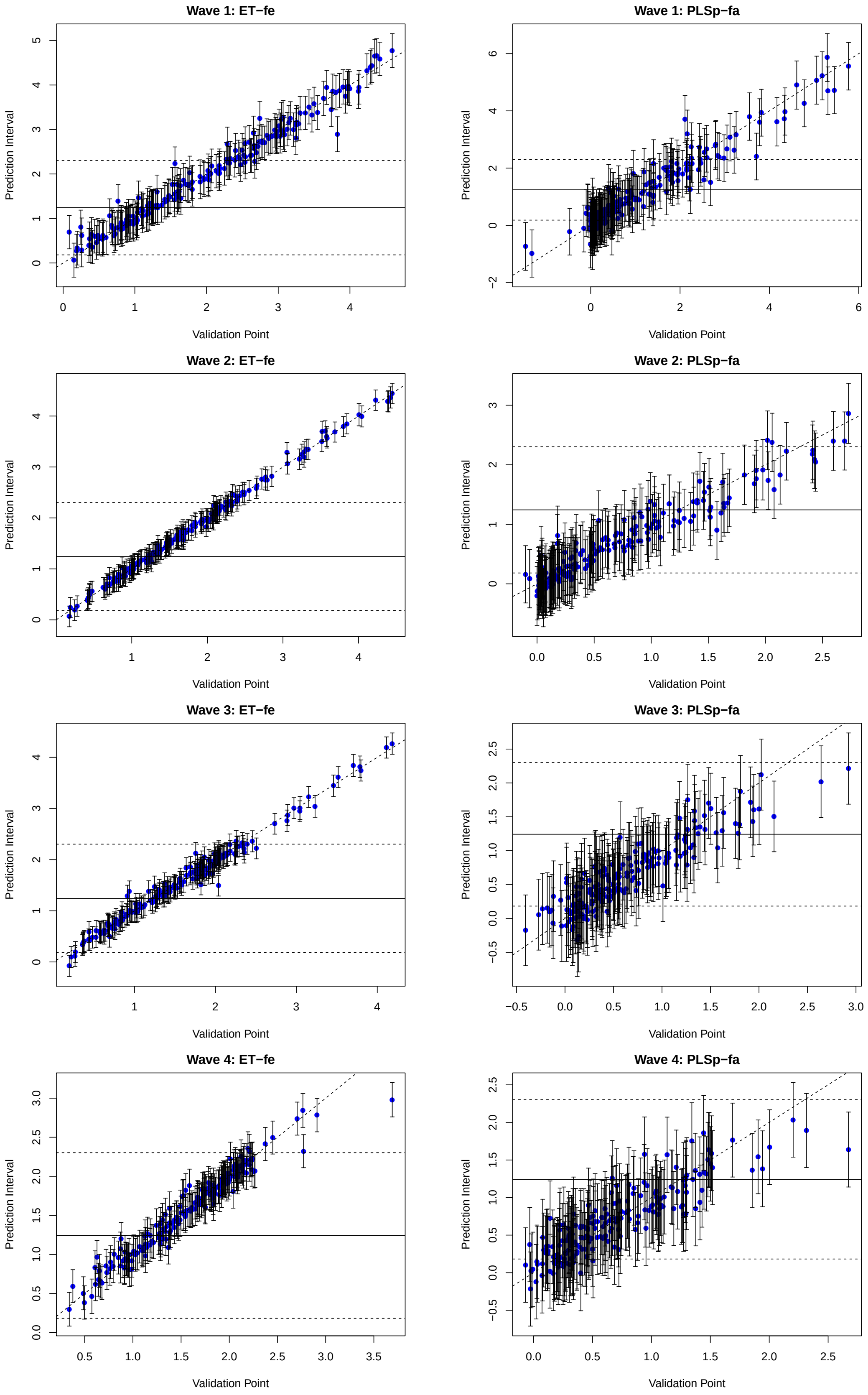

Table 18 Progression of the emulators for ET_fe (left column) and $PLSp_fa$ (right column) through waves 1 to 4 (top to bottom row). The horizontal lines give the target interval.

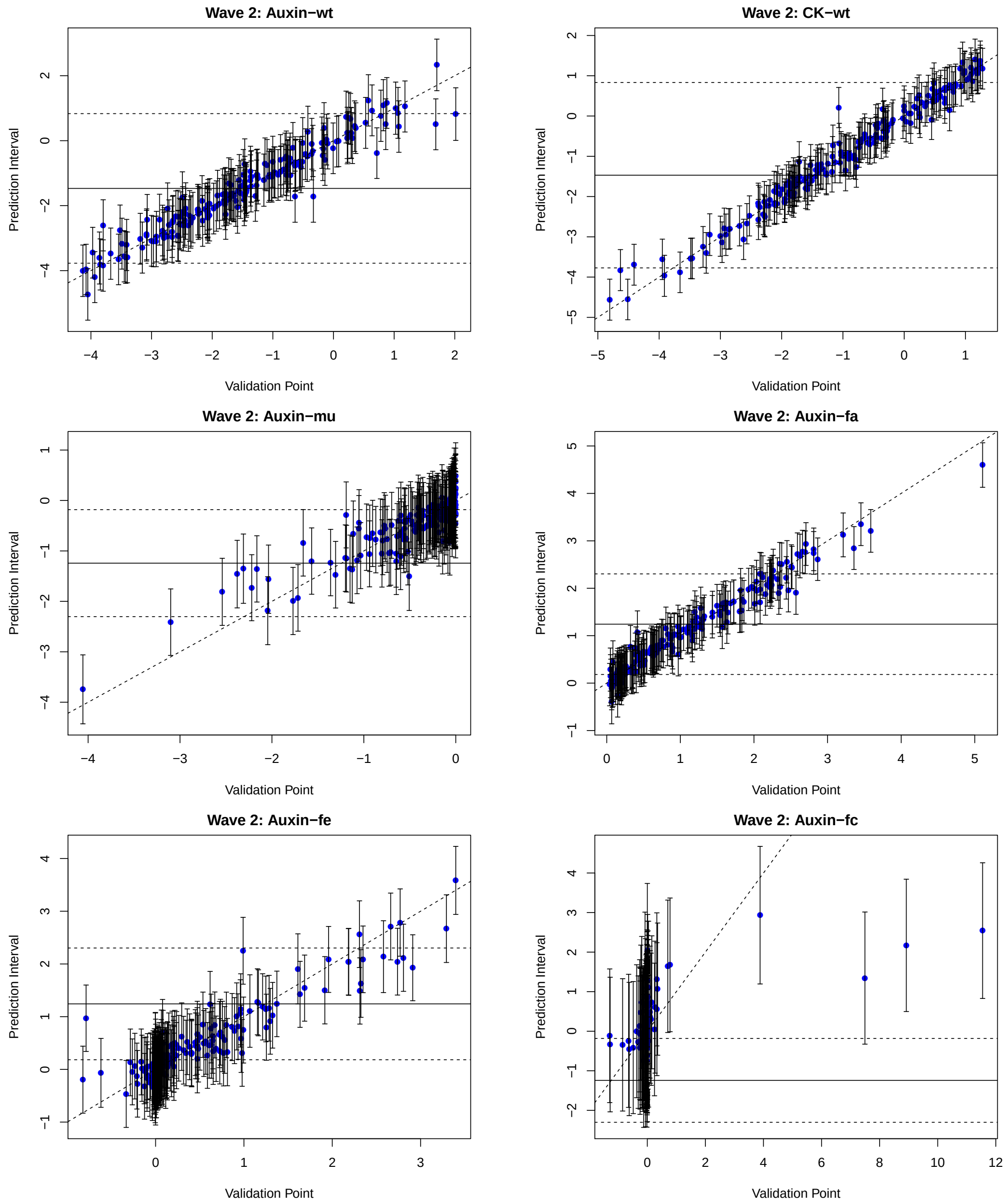

Table 19 Wave 2 emulator diagnostics for 6 out of 18 outputs considered. The horizontal lines give the target interval.

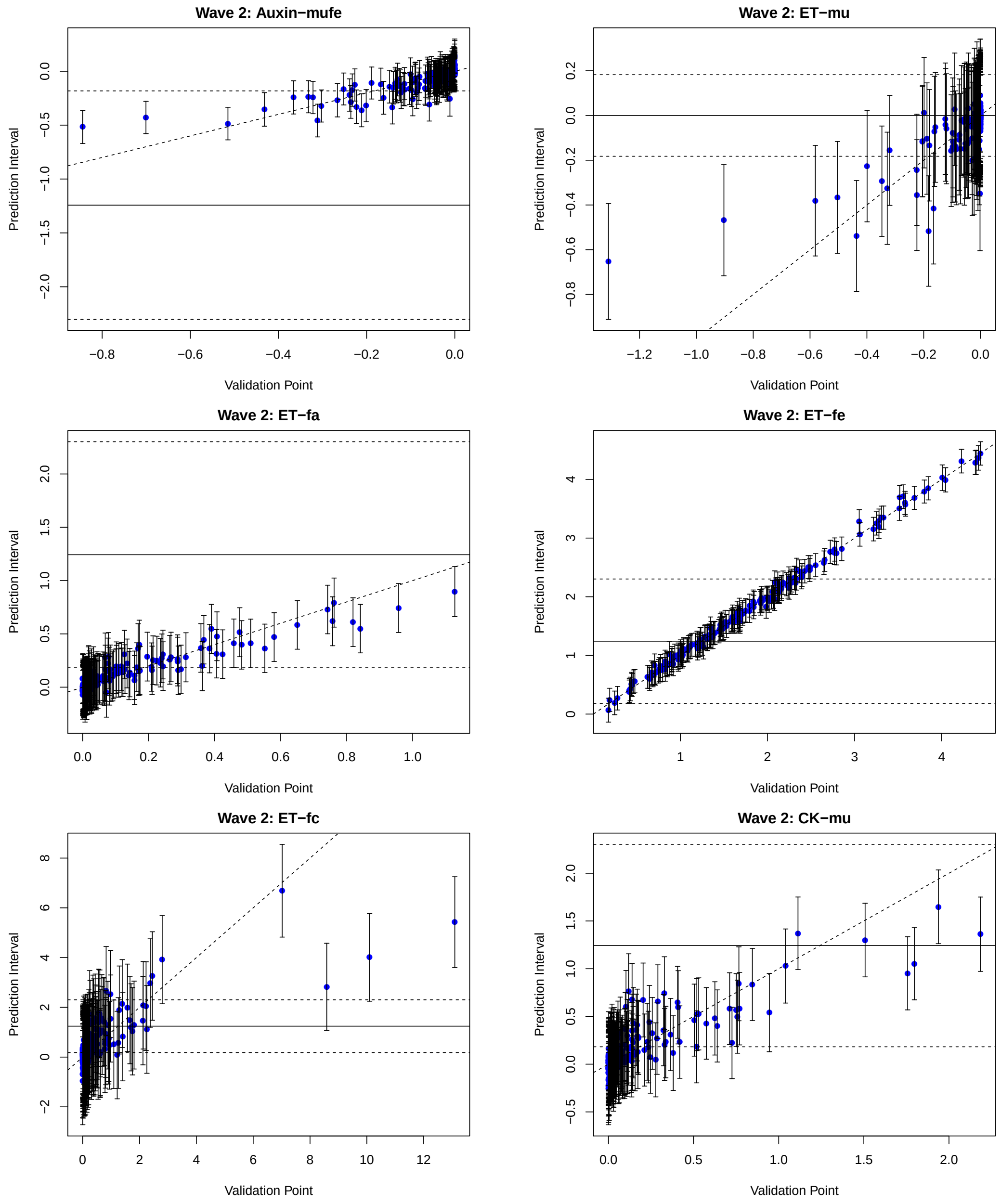

Table 20 Wave 2 emulator diagnostics for 6 out of 18 outputs considered. The horizontal lines give the target interval.

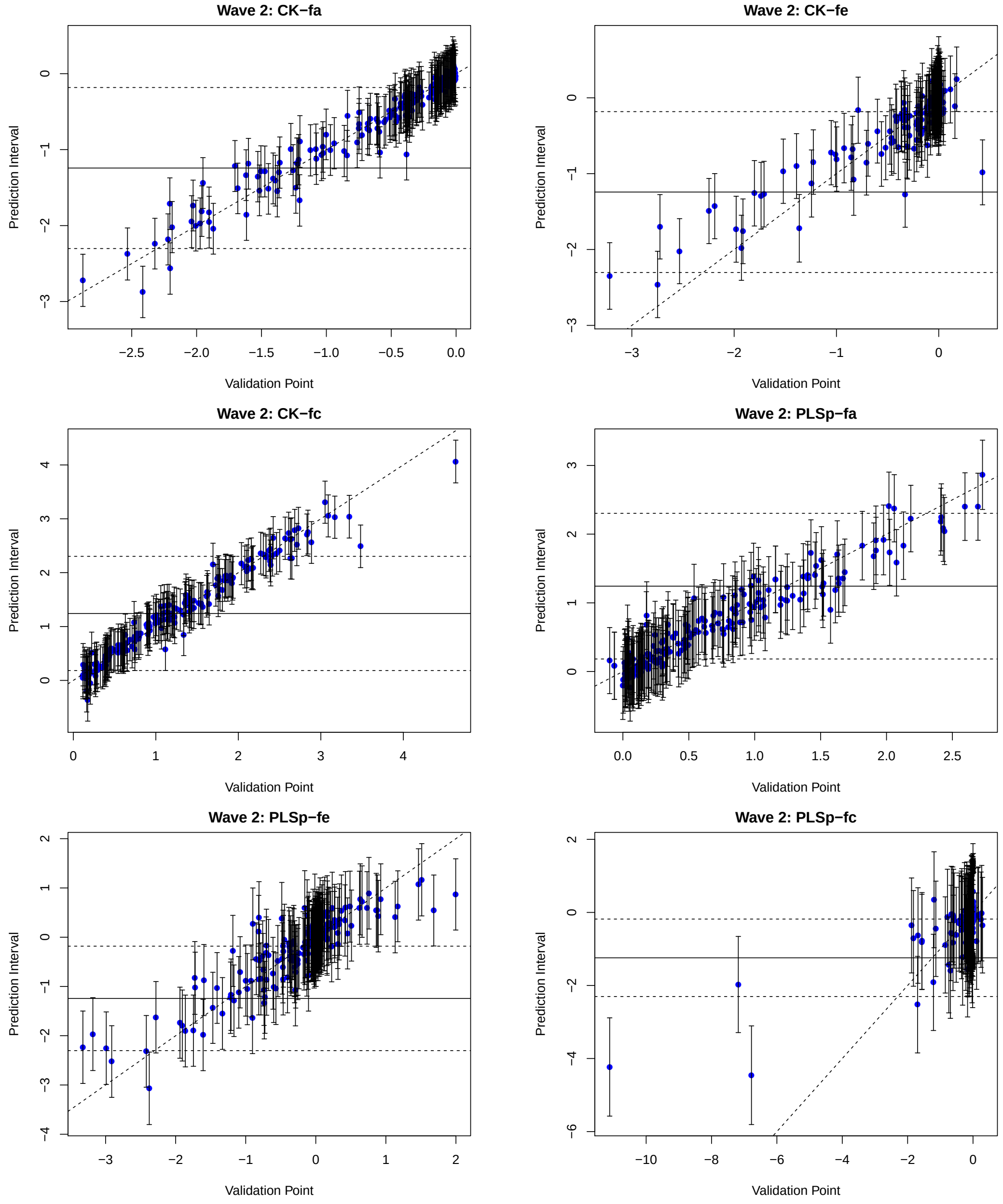

Table 21 Wave 2 emulator diagnostics for 6 out of 18 outputs considered. The horizontal lines give the target interval.

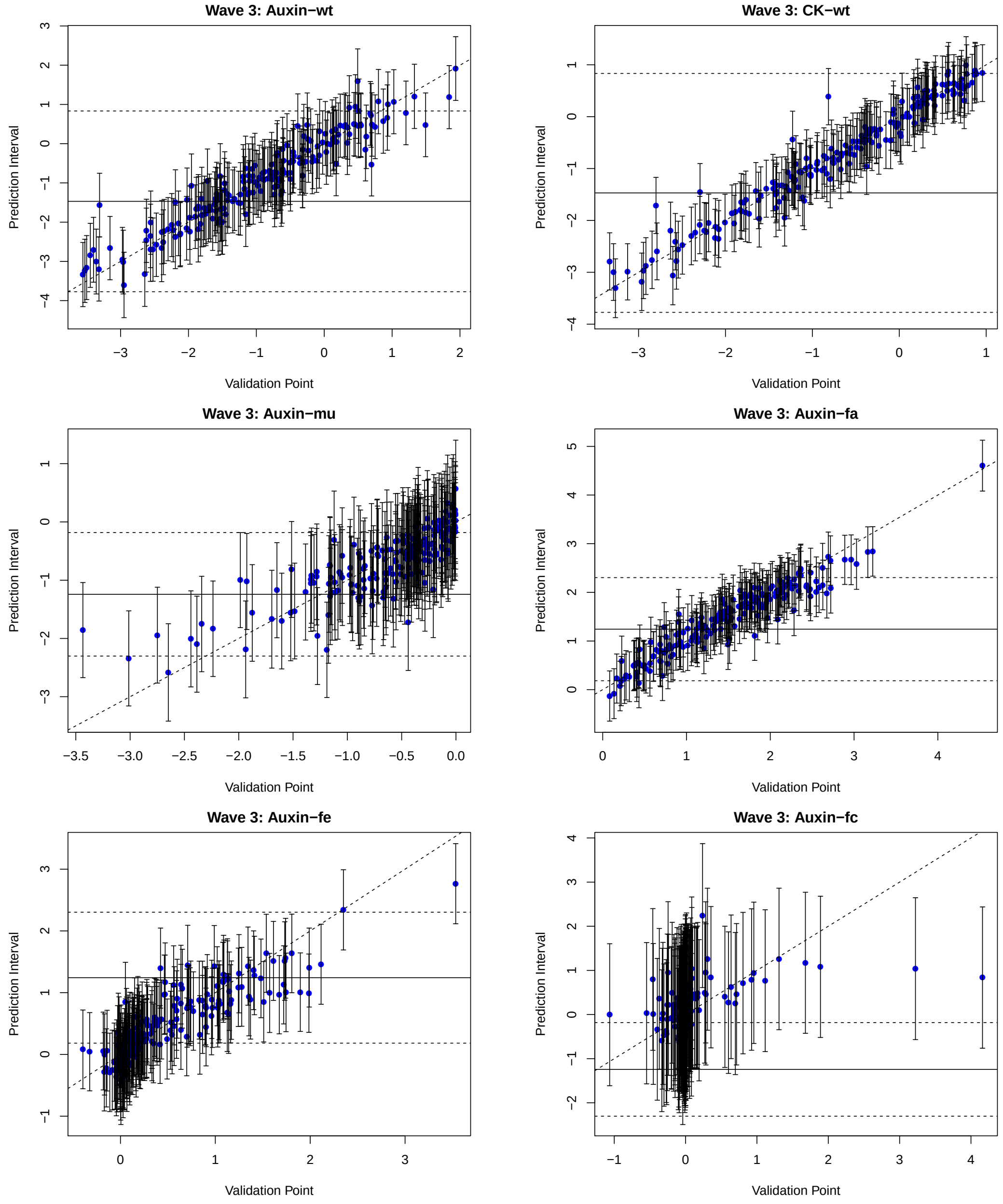

Table 22 Wave 3 emulator diagnostics for 6 out of 18 outputs considered. The horizontal lines give the target interval.

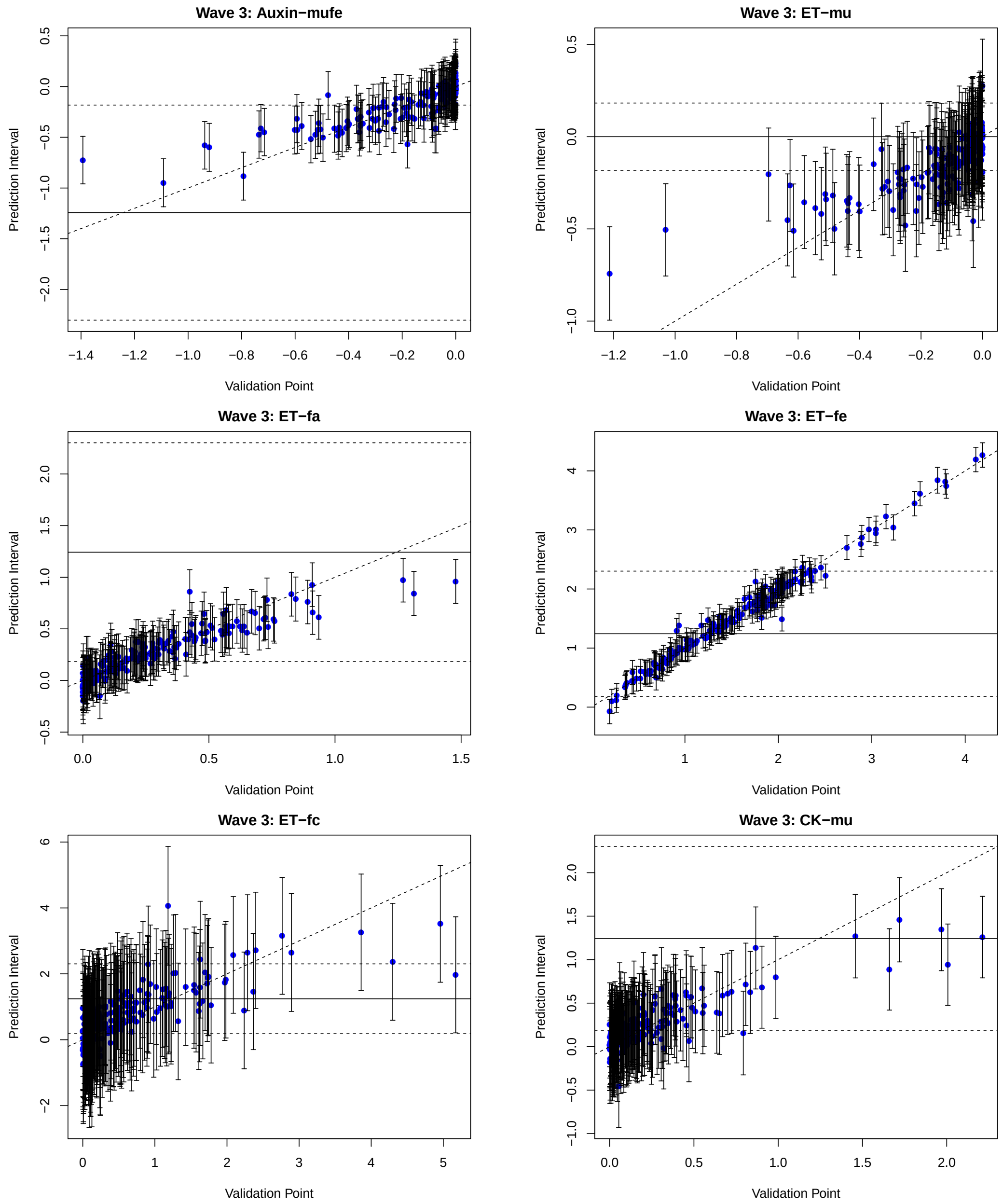

Table 23 Wave 3 emulator diagnostics for 6 out of 18 outputs considered. The horizontal lines give the target interval.

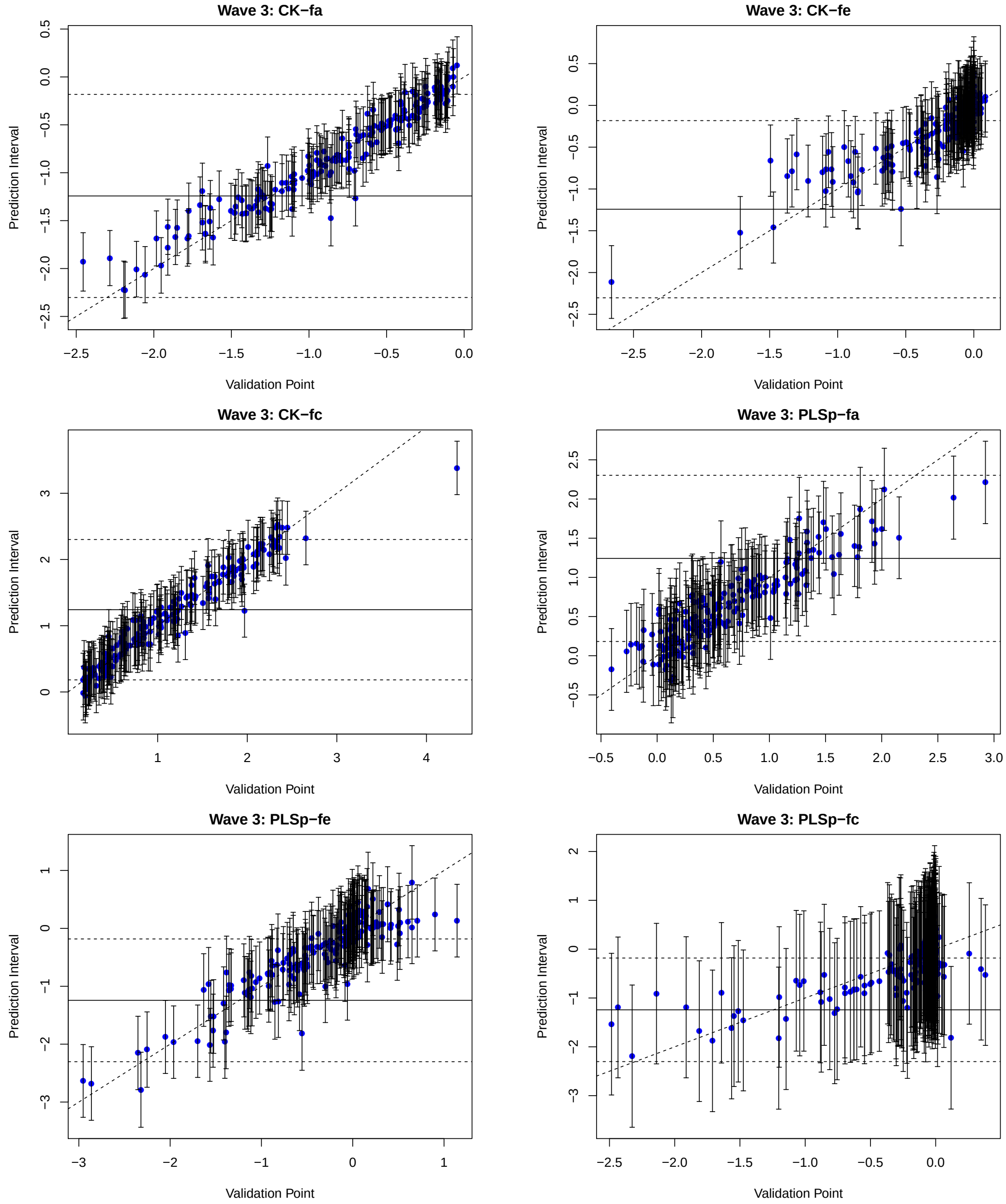

Table 24 Wave 3 emulator diagnostics for 6 out of 18 outputs considered. The horizontal lines give the target interval.

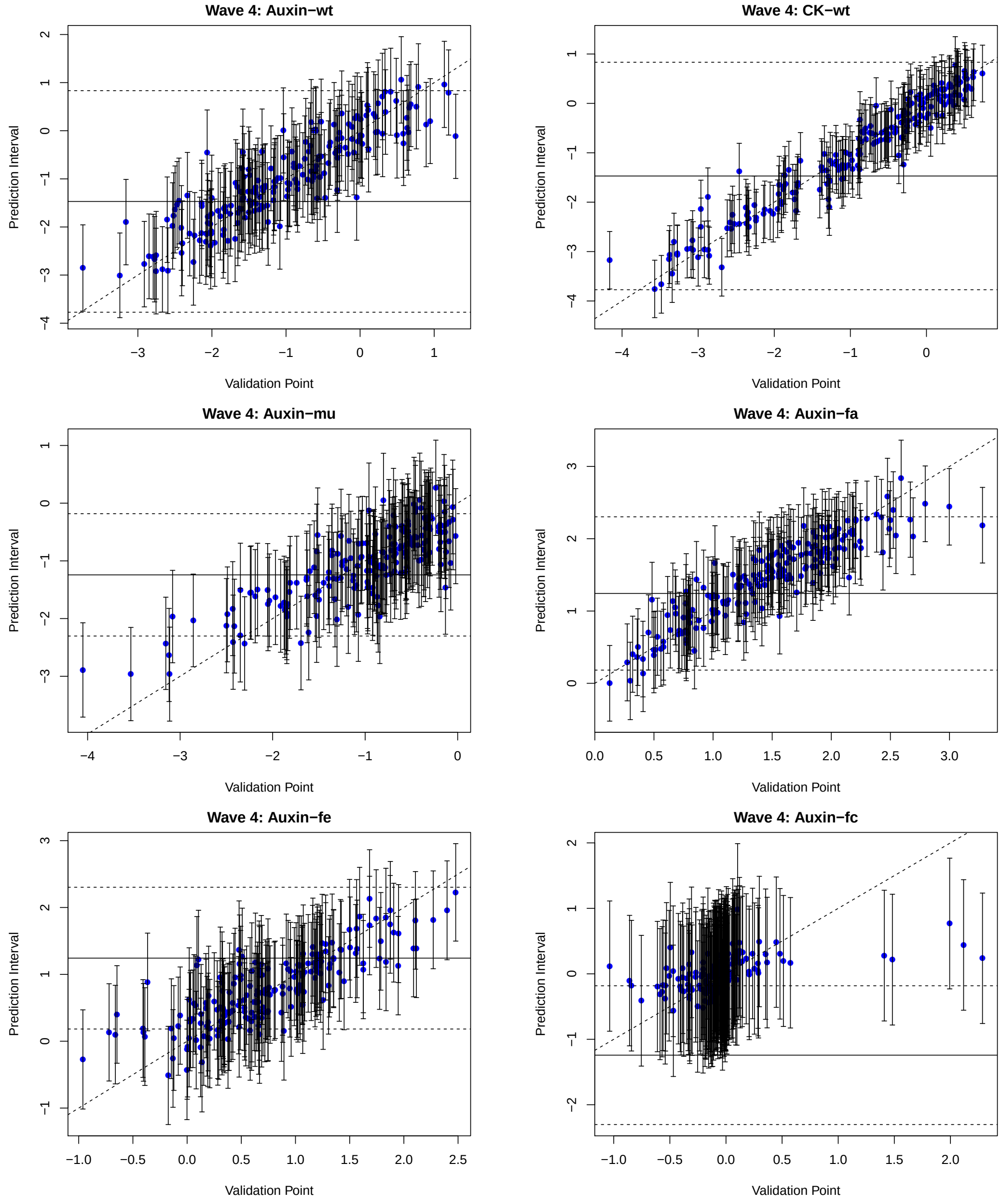

Table 25 Wave 4 emulator diagnostics for 6 out of 18 outputs considered. The horizontal lines give the target interval.

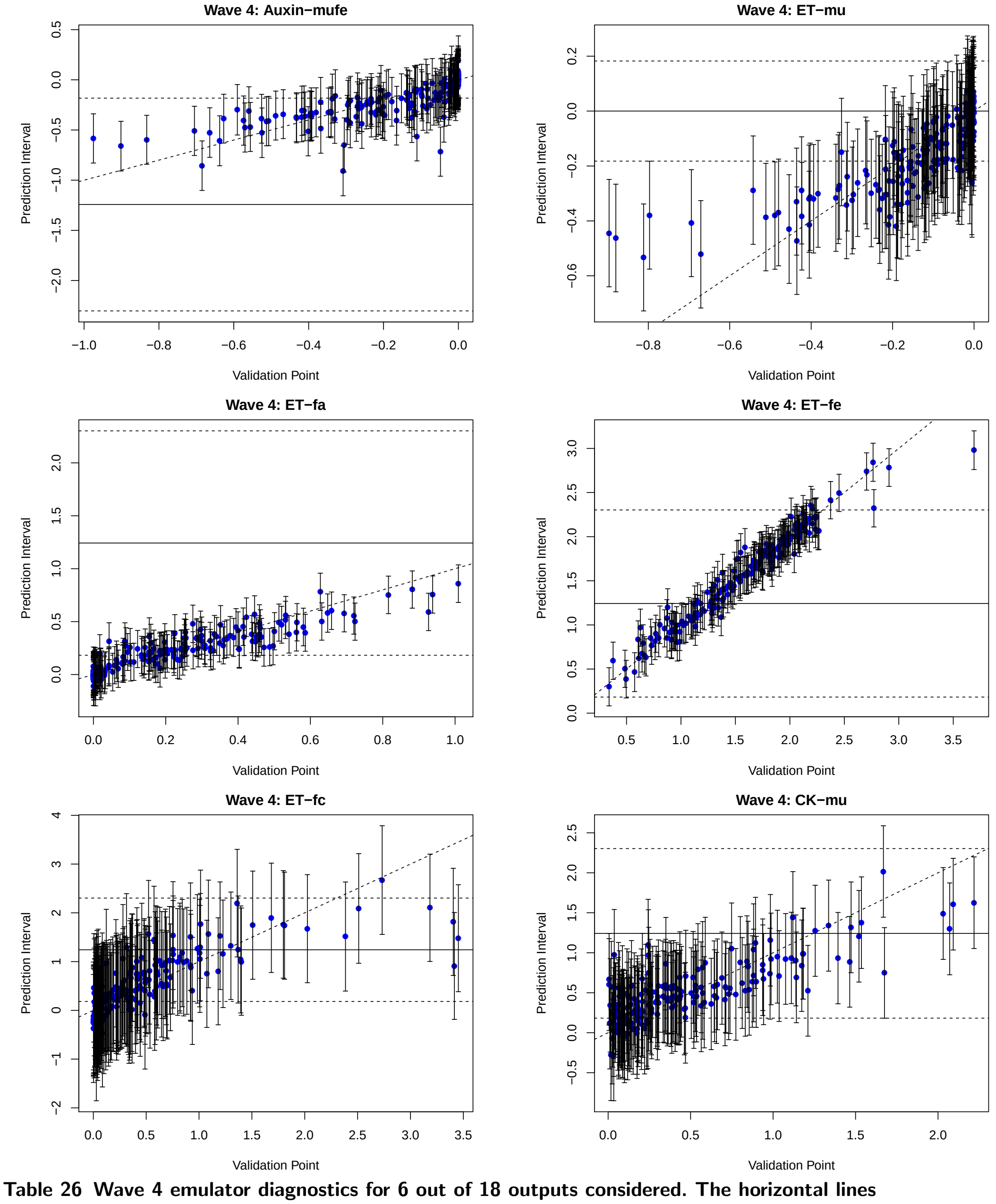

Table 26 Wave 4 emulator diagnostics for 6 out of 18 outputs considered. The horizontal lines give the target interval.

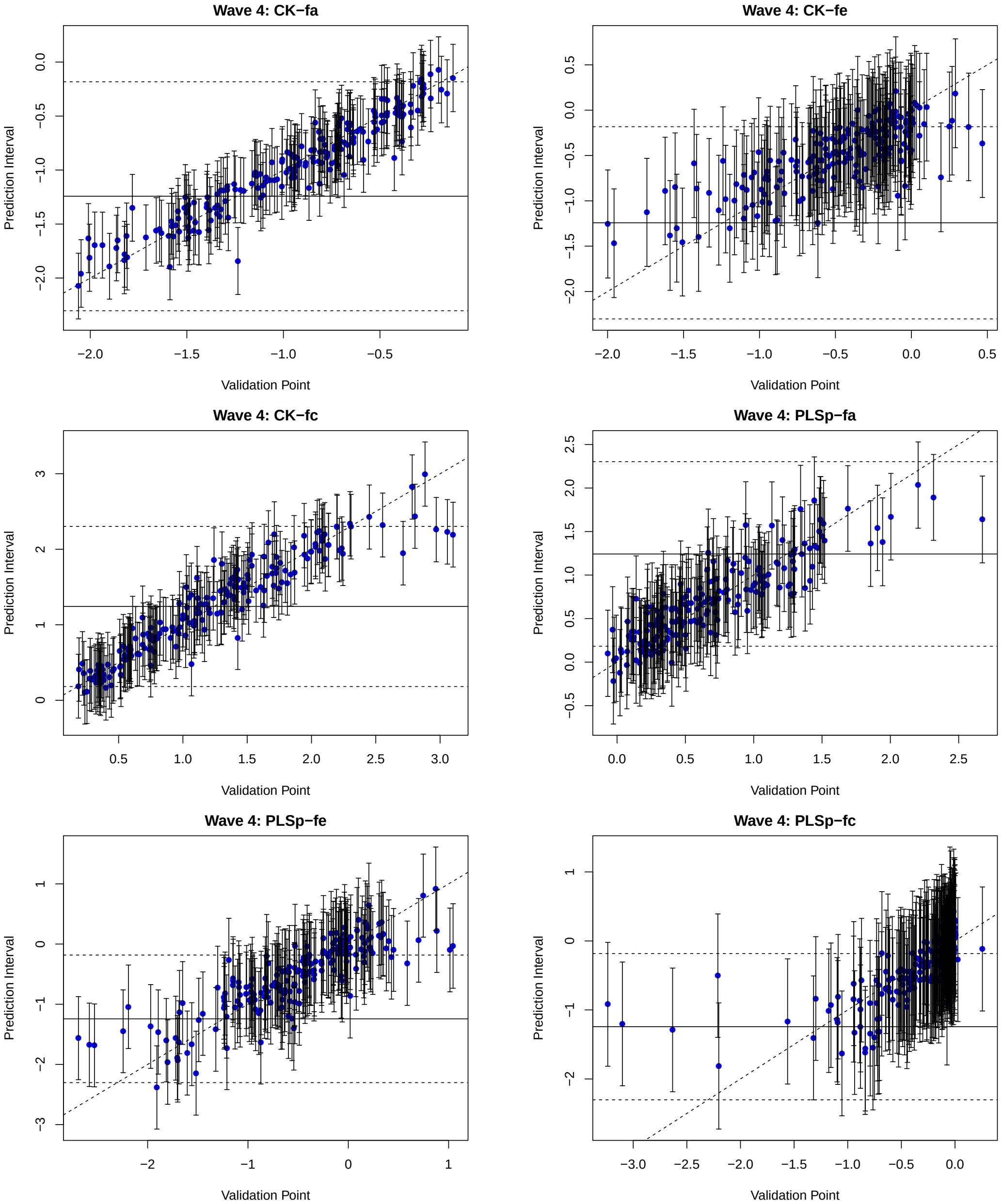

Table 27 Wave 4 emulator diagnostics for 6 out of 18 outputs considered. The horizontal lines give the target interval.
